# Supplementary material for: Structural and dynamic studies uncover a distinct allosteric modulatory site at the µ-opioid receptor
Source: Nat Commun. 2026 May 4;17:6000. doi: 10.1038/s41467-026-72633-z (PMC13347004; doi:10.1038/s41467-026-72633-z)
Supplement: Supplementary file 1 — Supplementary Information [file 41467_2026_72633_MOESM1_ESM.pdf]

Supplementary Information for

Structural and dynamic studies uncover a distinct allosteric modulatory site at the μ-opioid receptor

Haonan Zhang, Kirill Konovalov, Alexandra K. Parpounas, Davide Provasi, Shifan Yang, Alejandro Abraham, Aileen M. Vela, Audrey L. Warren, Gregory Zilberg, Suri Wang, Marta Filizola*, Daniel Wacker*

*To whom correspondence should be addressed: Marta Filizola (marta.filizola@mssm.edu), Daniel Wacker (Daniel.wacker@mssm.edu)

This file includes:

Supplementary Figures 1-8

Supplementary Tables 1-4

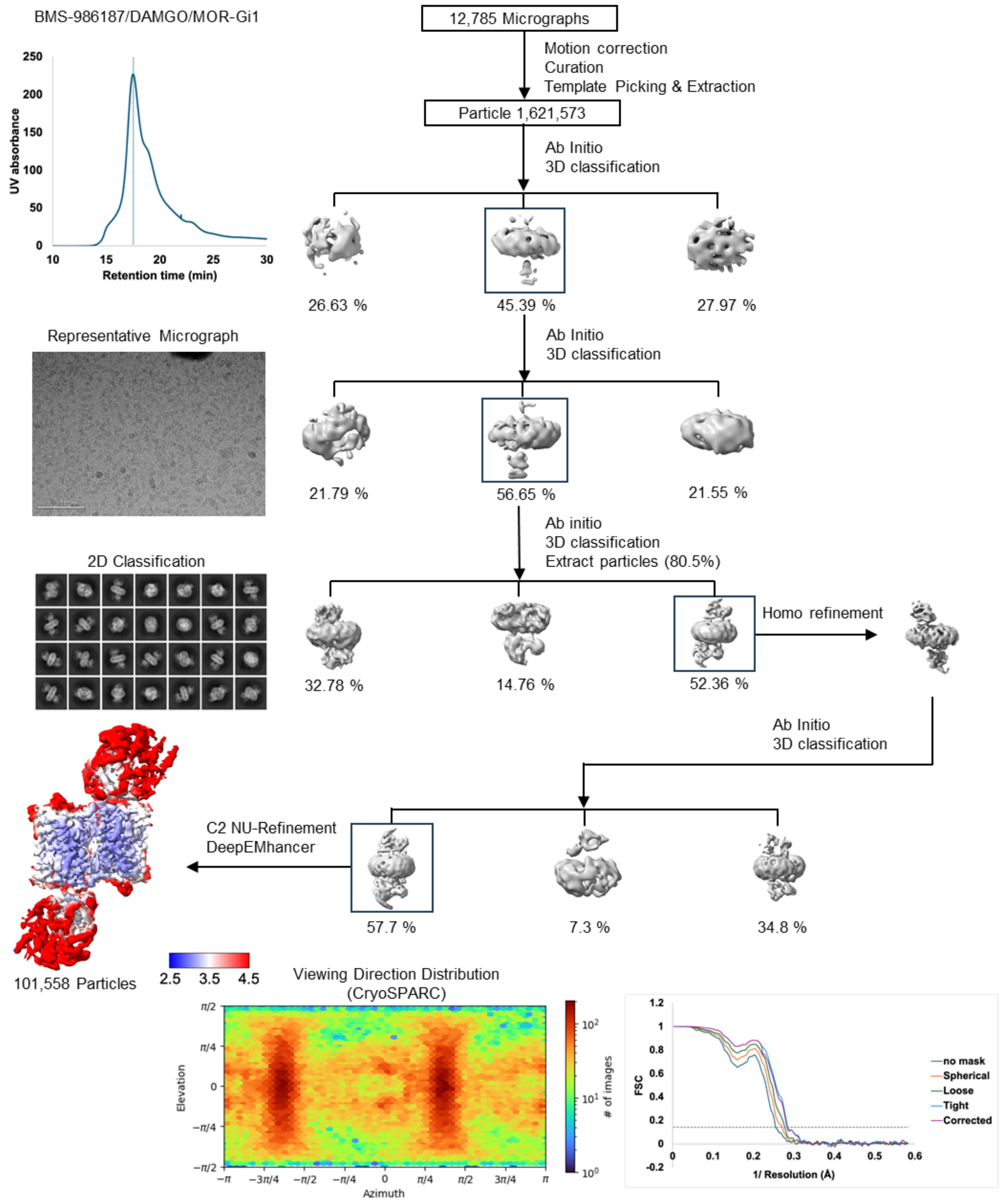

BMS-986187/DAMGO/MOR-Gi1
UV absorbance
Retention time (min)
Representative Micrograph
2D Classification
12,785 Micrographs
Motion correction
Curation
Template Picking & Extraction
Particle 1,621,573
Ab Initio
3D classification
26.63 %
45.39 %
27.97 %
Ab Initio
3D classification
21.79 %
56.65 %
21.55 %
Ab initio
3D classification
Extract particles (80.5%)
32.78 %
14.76 %
52.36 %
Homo refinement
Ab Initio
3D classification
C2 NU-Refinement
DeepEMhancer
57.7 %
7.3 %
34.8 %
101,558 Particles
2.5
3.5
4.5
Viewing Direction Distribution
(CryoSPARC)
Elevation
Azimuth
# of images
FSC
1/ Resolution (Å)
no mask
Spherical
Loose
Tight
Corrected

Supplementary Fig. 1 | Purification, and cryo-EM structure determination of the BMS-986187-bound DAMGO/MOR-Gi1 complex. Analytical size exclusion chromatogram shows largely monodisperse BMS-986187/DAMGO/MOR-Gi1 complex sample. Data were collected on a 300 keV Krios, a representative micrograph is shown, and processed in cryoSPARC v4.1.2.: Particles were picked from motion corrected micrographs, subjected to 2D classification (representative classes are shown), followed by ab initio model building and 3D classification. After multiple rounds of 3D classification, the final particle stack of 101,558 particles was subjected to non-uniform refinement applying C2 symmetry. A final map was obtained using DeepEMhancer and GS-FSC indicates a global resolution of 3.47 Å applying the 0.143 cutoff. Viewing direction distribution analysis (cryoSPARC) indicates sufficient coverage. Calculations in cryoSPARC indicate local resolutions of up to 3 Å around the BMS-986187 binding site. Source data are provided as a Source Data file.

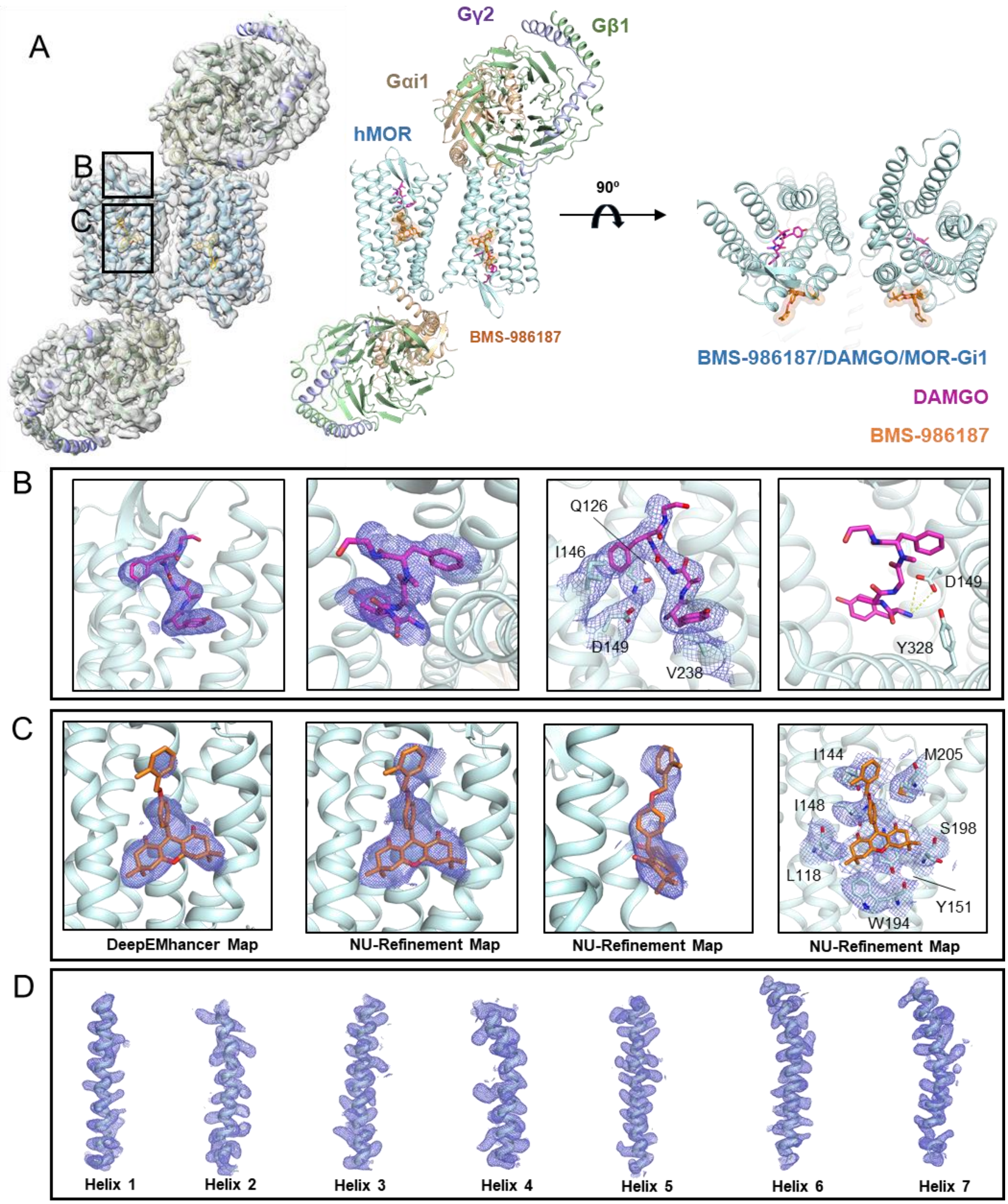
A
B
C
Gγ2
Gβ1
Gαi1
hMOR
90º
BMS-986187
BMS-986187/DAMGO/MOR-Gi1
DAMGO
BMS-986187
B
Q126
I146
D149
V238
D149
Y328
C
I144
M205
I148
S198
L118
Y151
W194
DeepEMhancer Map
NU-Refinement Map
NU-Refinement Map
NU-Refinement Map
D
Helix 1
Helix 2
Helix 3
Helix 4
Helix 5
Helix 6
Helix 7

Supplementary Fig. 2 | Representative map density of BMS-986187/DAMGO/MOR-Gi1 complex cryo-EM structure. (A) Overall cryo-EM map processed using DeepEMhancer and fitted with BMS-986187-bound DAMGO/MOR-Gi1 model built in COOT and refined in PHENIX. Inlays highlight map densities for DAMGO and surrounding residues (B), BMS-986187 and surrounding residues (C), and representative areas of MOR (D). MOR, DAMGO, and BMS-986187 are shown in palecyan, magenta, and orange, respectively. Densities are shown as blue mesh with contour levels of 4.0σ for the DeepEMhancer map of DAMGO (B), 3.0σ for the DeepEMhancer map of cholesterol and BMS-986187, and 3.5σ for the NU-refinement map of BMS-986187 (C), as well as 3.4σ for the DeepEMhancer map of representative areas of MOR (D).

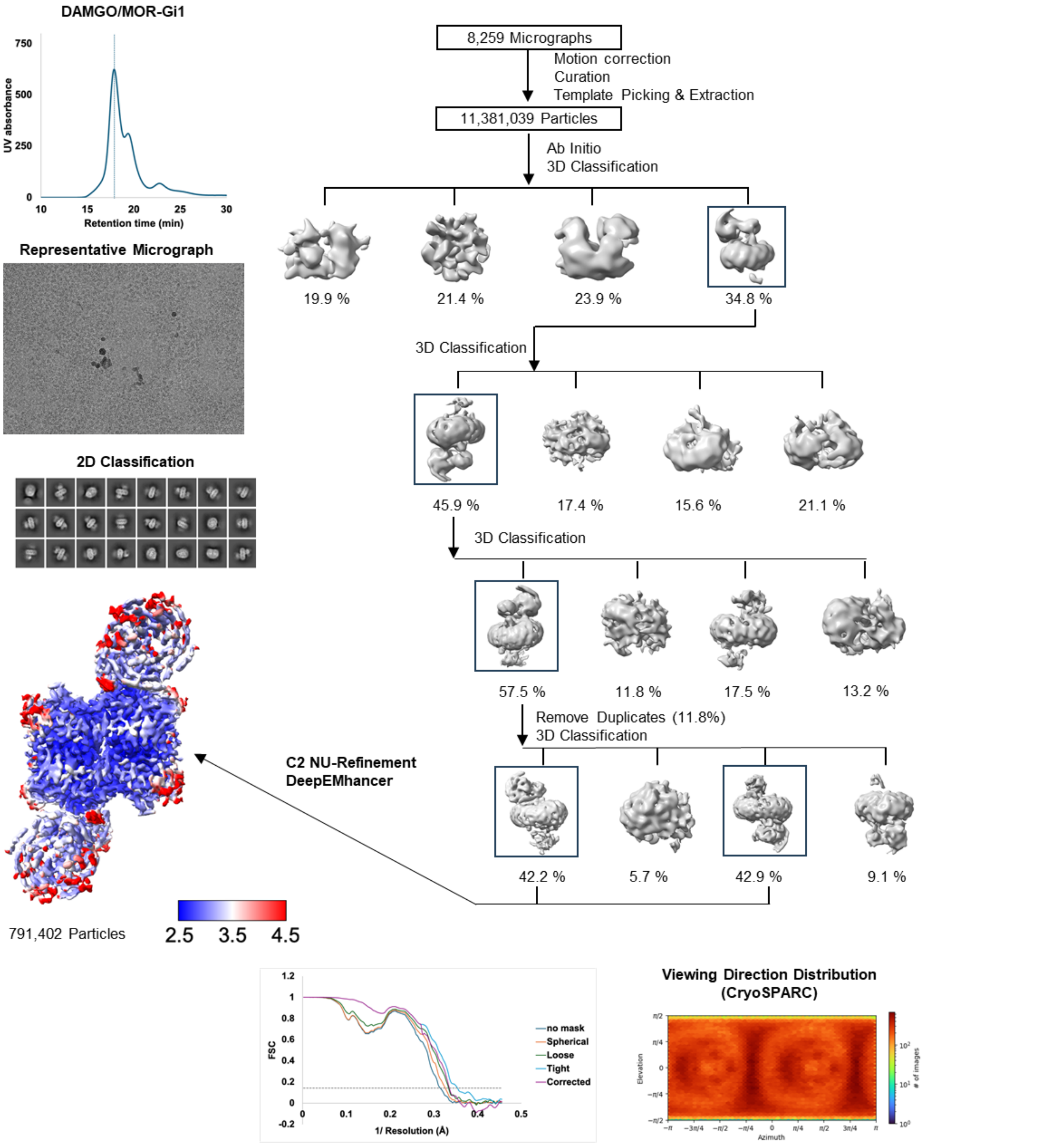
DAMGO/MOR-Gi1
UV absorbance
Retention time (min)
Representative Micrograph
2D Classification
8,259 Micrographs
Motion correction
Curation
Template Picking & Extraction
11,381,039 Particles
Ab Initio
3D Classification
19.9 %
21.4 %
23.9 %
34.8 %
3D Classification
45.9 %
17.4 %
15.6 %
21.1 %
3D Classification
57.5 %
11.8 %
17.5 %
13.2 %
Remove Duplicates (11.8%)
3D Classification
42.2 %
5.7 %
42.9 %
9.1 %
C2 NU-Refinement
DeepEMhancer
791,402 Particles
2.5
3.5
4.5
FSC
1/ Resolution (Å)
no mask
Spherical
Loose
Tight
Corrected
Viewing Direction Distribution
(CryoSPARC)
Elevation
Azimuth
# of images

Supplementary Fig. 3 | Purification, and cryo-EM structure determination of the PAM-free DAMGO/MOR-Gi1 complex. Analytical size exclusion chromatogram shows largely monodisperse DAMGO/MOR-Gi1 complex sample. Data were collected on a 300 keV Krios, a representative micrograph is shown, and processed in cryoSPARC v4.1.2.: Particles were picked from motion corrected micrographs, subjected to 2D classification (representative classes are shown), followed by ab initio model building and 3D classification. After multiple rounds of 3D classification, the final particle stack of 791,402 particles was subjected to non-uniform refinement applying C2 symmetry. A final map was obtained using DeepEMhancer and GS-FSC indicates a global resolution of 2.89 Å applying the 0.143 cutoff. Viewing direction distribution analysis (cryoSPARC) indicates sufficient coverage. Calculations in cryoSPARC indicate local resolutions of up to 2.5 Å around the BMS-986187 binding site. Source data are provided as a Source Data file.

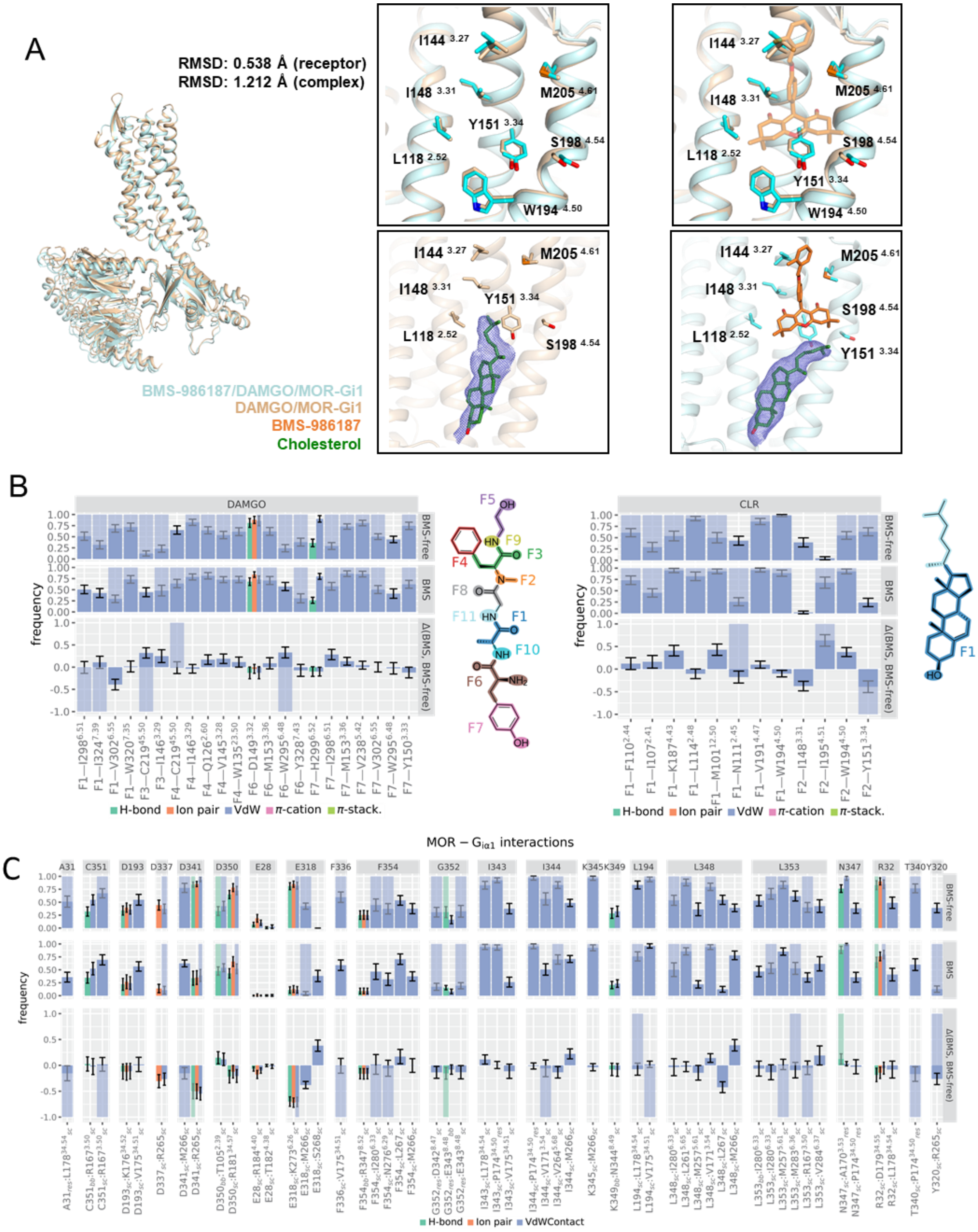
A
RMSD: 0.538 Å (receptor)
RMSD: 1.212 Å (complex)
BMS-986187/DAMGO/MOR-Gi1
DAMGO/MOR-Gi1
BMS-986187
Cholesterol
B
DAMGO
CLR
BMS-free
BMS
Δ(BMS, BMS-free)
frequency
H-bond
Ion pair
VdW
π-cation
π-stack.
C
MOR – Giα1 interactions
H-bond
Ion pair
VdWContact

Supplementary Fig. 4 | Effect of BMS-986187 on interactions within the MOR signaling complex. (A) Comparison of BMS-986187-bound (palecyan) and PAM-free (wheat) DAMGO/MOR-Gi1 cryo-EM structures. Top panel shows zoom in of BMS-986187 binding site and key residues, and bottom panel highlights conformational shift of cholesterol upon BMS-986187 binding. Densities for cholesterol are shown as blue mesh with a contour level of 4.0σ (PAM-free state) and 3.0σ (BMS-986187-bound state). (B–D) Comparison of interaction frequencies between DAMGO and MOR as well as cholesterol (CLR) and MOR (B), and interaction frequencies between Gαi1 and MOR (C) in molecular dynamics (MD) simulations with and without BMS-986187. Bar height represents the average interaction frequency across the trajectory, with error bars indicating the standard deviation calculated over 25 ns time blocks. Blue shading over a bar denotes interactions also observed in the cryo-EM structure. Each panel contains three facets: the top two show interaction frequencies in BMS-free and BMS-bound simulations, respectively; the bottom facet shows the difference in interaction frequency between the two conditions. Molecular fragmentations of DAMGO and CLR used for grouping interaction data are shown in B.

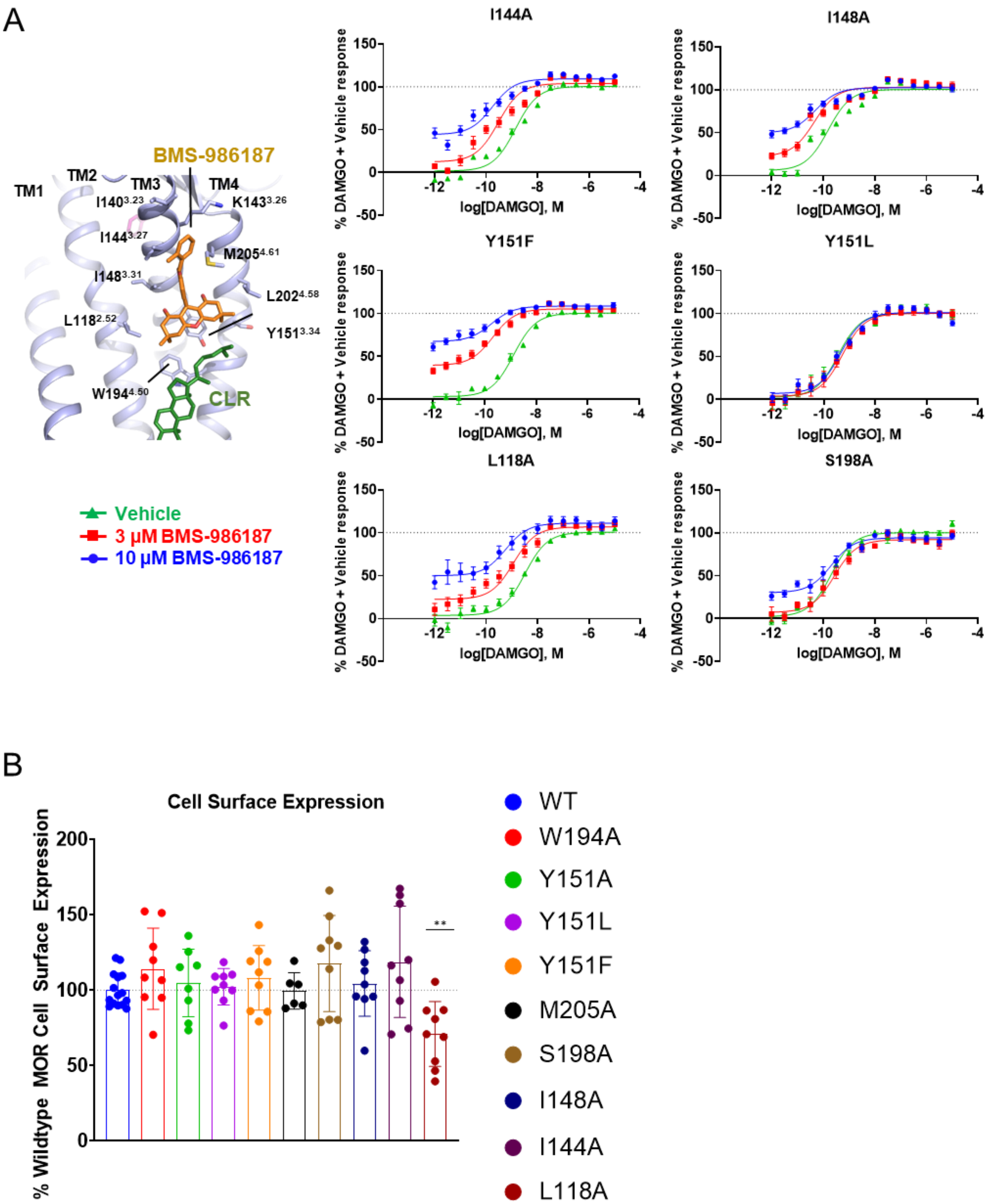
A
BMS-986187
TM1
TM2
TM3
TM4
I140^3.23
K143^3.26
I144^3.27
M205^4.61
I148^3.31
L202^4.58
L118^2.52
Y151^3.34
W194^4.50
CLR
Vehicle
3 µM BMS-986187
10 µM BMS-986187
I144A
I148A
Y151F
Y151L
L118A
S198A
% DAMGO + Vehicle response
log[DAMGO], M
B
Cell Surface Expression
% Wildtype MOR Cell Surface Expression
**
WT
W194A
Y151A
Y151L
Y151F
M205A
S198A
I148A
I144A
L118A

Supplementary Fig. 5 | Effect of site directed receptor mutations on BMS-986187-modulated DAMGO activity at MOR as measured by cAMP accumulation. (A) Zoom in on BMS-986187 and key residues in the PAM binding site and DAMGO concentration response data in the presence of vehicle (DMSO, green), 3 µM BMS-986187 (red), and 10 µM BMS-986187 (green). (B) Cell surface expression of wildtype and mutant constructs as determined by ELISA. All data from signaling and expression studies represent mean ± SEM of three to five independent experiments (n = 3-5) performed in triplicate, and have been normalized to the DAMGO + vehicle response or expression of wildtype MOR, respectively. One-way ANOVA using Fisher's least significance difference post hoc test was used to determine significance of differences between construct cell surface expression compared to wildtype MOR, with only the L118A mutant showing statistically significant differences (P=0.0034). Source data are provided as a Source Data file.

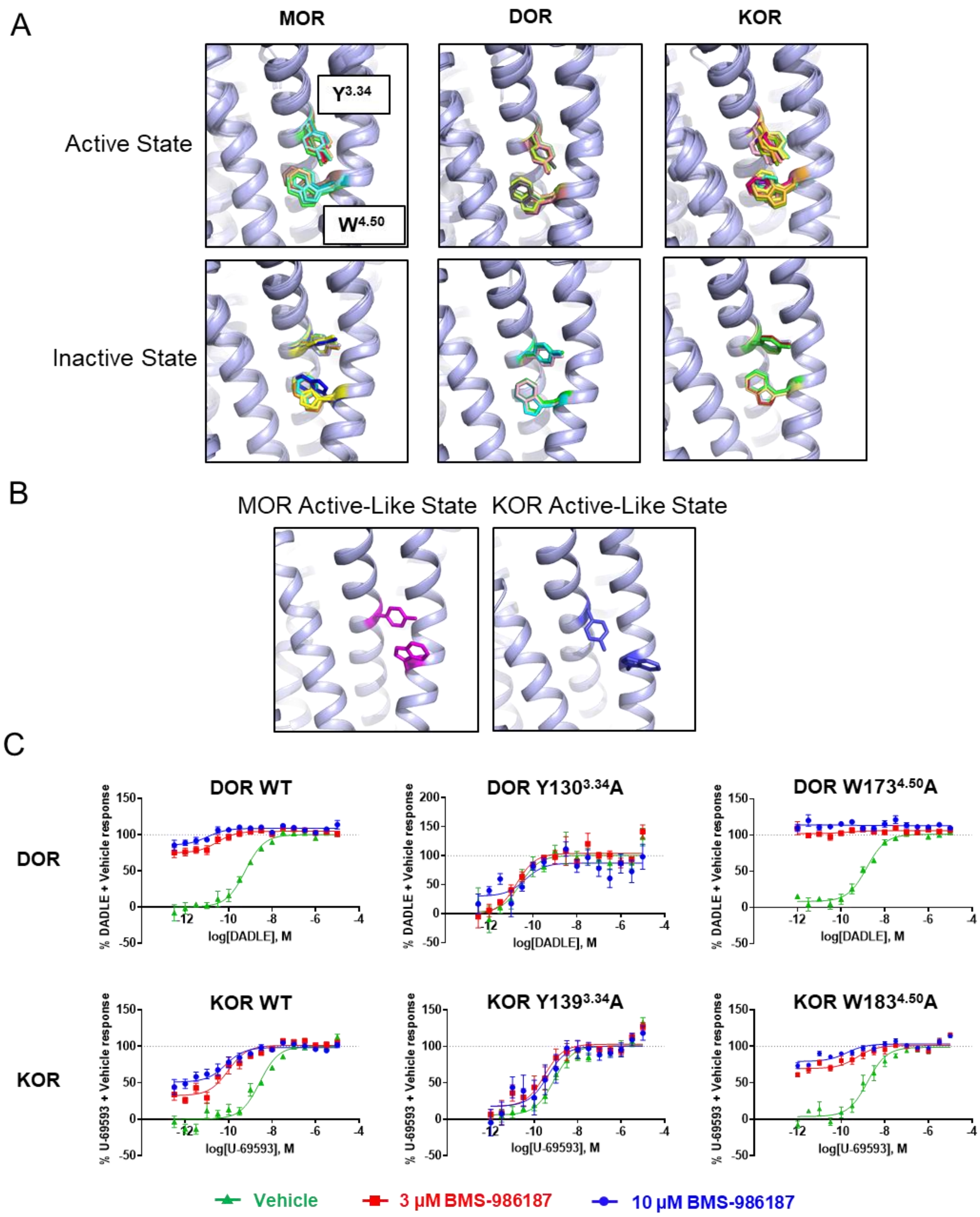
A
MOR
DOR
KOR
Active State
Inactive State
Y3.34
W4.50
B
MOR Active-Like State
KOR Active-Like State
C
DOR
KOR
DOR WT
DOR Y130^3.34A
DOR W173^4.50A
KOR WT
KOR Y139^3.34A
KOR W183^4.50A
% DADLE + Vehicle response
log[DADLE], M
% U-69593 + Vehicle response
log[U-69593], M
Vehicle
3 μM BMS-986187
10 μM BMS-986187

Supplementary Fig. 6 | Structural and functional analysis of $Y^{3.34}$ and $W^{4.50}$ conformations across different MOR, DOR, and KOR activation states. (A) Structures of inactive- and active-state opioid receptor structures (light blue) shown in the left and right columns respectively. $Y^{3.34}$ and $W^{4.50}$ are highlighted as sticks, and have been colored by structure (*MOR Inactive State Structures* – PDB: 4DKL (light blue), PDB: 7UL4 (limon), PDB: 8QOT (pale yellow), PDB: 9BJK (dark blue), PDB: 9MQH (yellow), PDB: 9MQI (orange), PDB: 9MDJ (cyan); *MOR Active State Structures* – PDB: 6DDE (green), PDB: 6DDF (red), PDB: 7SBF (wheat), PDB: 7SCG (light teal), PDB: 7T2G (dark salmon), PDB: 7T2H (pale green), PDB: 7U2K (light orange), PDB: 7U2L (purple), PDB: 8EF5 (brown), PDB: 8EF6 (forest green), PDB: 8EFB (dark red), PDB: 8EFL (pink), PDB: 8EFO (olive), PDB: 8EFQ (pale cyan), PDB: 8F7R (light pink), PDB: 8K9L (green cyan); *DOR Inactive State Structures* – PDB: 4EJ4 (aquamarine); PDB: 4N6H (green); PDB: 4RWA (light salmon); PDB: 4WRD (marine); *DOR Active State Structures* – PDB: 6PT2 (mint green), PDB: 6PT3 (light orange), PDB: 8F7S (salmon), PDB: 8Y45 (grey), PDB: 9CGJ (violet), PDB: 9CGK (limon); *KOR Inactive State Structures* – PDB: 4DJH (light blue), PDB: 6VI4 (green), PDB: 9MQK (yellow orange), PDB: 9MQL (dark red); *KOR Active State Structures* – PDB: 7YIT (pink), PDB: 8DZP (pale yellow), PDB: 8DZQ (bright orange), PDB: 8DZR (chartreuse), PDB: 8DZS (salmon), PDB: 8F7W (light orange), PDB: 8VVE (violet), PDB: 8VVF (light salmon), PDB: 8VVG (lime green), PDB: 9D61 (brown)). (B) MOR (PDB: 5C1M) and KOR (PDB: 6B73) nanobody-stabilized active-like state structures, with key residues colored in magenta and purple, respectively. (C) Allosteric effects of BMS-986187 on U-69593 signaling at wildtype and mutant KOR, as well as on DADLE signaling at wildtype and mutant DOR, determined in cAMP accumulation assays. Data from signaling studies represent mean ± SEM of two independent experiments ($n = 2$) performed in triplicate, and have been normalized to the U-69593 + vehicle and DADLE + vehicle response, respectively. Source data are provided as a Source Data file.

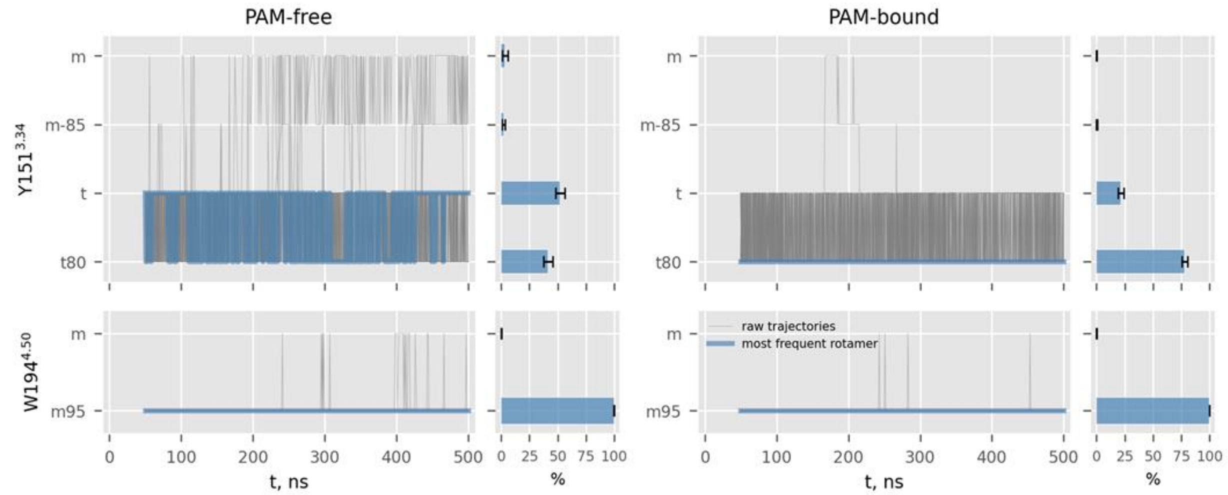

Supplementary Fig. 7 | Rotameric dynamics of Y151$^{3.34}$ and W194$^{4.50}$ in DAMGO/MOR–Gi1 simulations with and without BMS-986187. Time-resolved rotamer assignments (left panels) and corresponding population analyses (right panels) for the side chains of Y151$^{3.34}$ (left) and W194$^{4.50}$ (right) in PAM-free (top) and PAM-bound (bottom) DAMGO/MOR–Gi1 simulations. Rotameric states are classified as t, t80, m, m-85, and m95 according to the penultimate rotamer library by Lovell et al[84]. In this nomenclature, t denotes the trans conformation (≈180°) and m the gauche conformation (≈-60°) when χ angles cluster near these canonical values. When χ angles deviate from these ideal positions, the assigned state corresponds to the nearest 10° increment.. Data are shown for 20 independent 500 ns-trajectories after discarding the first 50 ns. Grey traces indicate raw rotamer assignments along individual trajectories, while the blue line denotes the most frequent rotamer at each time point over the 20 independent trajectories. Bar plots report the average percentage occupancy of each rotamer state, with confidence intervals given by the standard deviations estimated by bootstrapping individual trajectories. Y151$^{3.34}$ samples both t and t80 rotameric states in the PAM-free simulations, with minor populations of m-85 and m states. In contrast, binding of BMS-986187 stabilizes Y151$^{3.34}$ predominantly in the t80 rotamer, with only a small population of the t rotamer. In contrast, W194$^{4.50}$ remains mostly confined to a single m95 rotamer in both conditions, with a marginal exploration of other m states, indicating limited side-chain flexibility and minimal sensitivity to PAM binding.

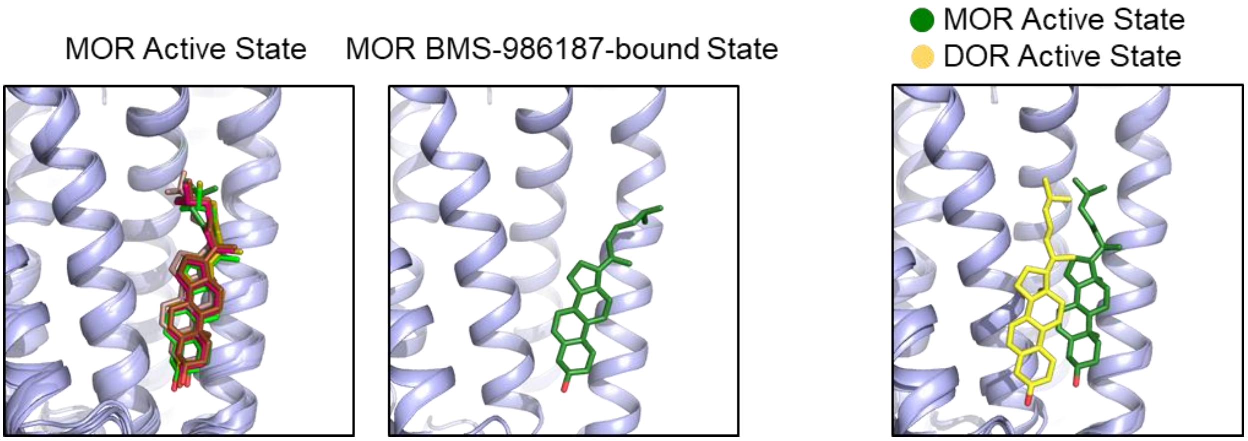

Supplementary Fig. 8 | Differences in cholesterol binding at different opioid receptors. Cholesterol binding poses in MOR active state structures (PDB: 7T2G (dark salmon), PDB: 8EF5 (brown), PDB: 8EF6 (green), PDB: 8EFL (pink), PDB: 8EFO (olive), PDB: 9PUD (forest green)) and the BMS-986187-bound MOR active state (PDB: 9PU5 (forest green)), in the left and middle panels respectively. Comparison of cholesterol binding poses in active state structures of DOR (PDB: 8F7S (yellow)) and MOR (PDB: 9PUD (forest green)) shown in the right panel.

Supplementary Table 1 | Cryo-EM data collection, refinement and validation statistics.

	**BMS986187/ hMOR-Gi1-DAMGO** (EMD-71869) (PDB ID: 9PU5)	**BMS986187/ hMOR-DAMGO** (EMD-71869) (PDB ID: 10TL)	**hMOR-Gi1-DAMGO** (EMD-71871) (PDB ID: 9PUD)	**hMOR-DAMGO** (EMD-71871) (PDB ID: 10TM)
**Data collection and processing**				
Magnification	105,000	105,000	64,000	64,000
Voltage (kV)	300	300	300	300
Electron exposure (e–/Å^2)	50.74	50.74	51.94	51.94
Defocus range (μm)	-0.7 ~ -2.2	-0.7 ~ -2.2	-0.8 ~ -2.0	-0.8 ~ -2.0
Pixel size (Å)	0.844	0.844	1.076	1.076
Symmetry imposed	C2	C2	C2	C2
Initial particle images (no.)	7,018,231	7,018,231	12,494,868	12,494,868
Final particle images (no.)	101,558	101,558	791,402	791,402
Map resolution (Å)	3.47	3.47	2.89	2.89
FSC threshold	0.143	0.143	0.143	0.143
Map sharpening B-factor (Å^2)	-152.8	-152.8	-140.9	-140.9
**Refinement**				
Model composition				
Non-hydrogen atoms	14466	4836	14284	4654
Protein residues	1808	564	1808	564
Ligands	2	2	1	1
R.m.s. deviations				
Bond lengths (Å)	0.012	0.020	0.007	0.007
Bond angles (°)	1.035	0.991	1.128	0.984
Validation				
Clashscore	8.07	4.97	14.29	12.45
Poor rotamers (%)	0	0	0.26	0
Ramachandran plot				
Favored (%)	98.21%	97.5%	95.58%	96.79%
Allowed (%)	1.68%	2.50%	4.31%	3.21%
Disallowed (%)	0.11	0	0.11	0

Supplementary Table 2 | Potencies and efficacies of DAMGO in MOR signaling assays using wildtype and mutant MOR in the presence and absence of different BMS-986187 concentrations. Data represent mean EC50 or pEC50 ± SEM, efficacy as span ± SEM, and intrinsic efficacy of BMS-986187 calculated as the difference in spans between DAMGO concentration responses in the presence of vehicle or PAM. Biological repeats for each dataset are indicated, and all experiments were performed in triplicate. ND = Not determined. Source data are provided as a Source Data file.

cAMP Accumulation

MOR Construct	Replicates	DMSO		DAMGO		BMS-986187	
		EC50 (nM)<br>pEC50±SEM	Intrinsic Efficacy,<br>(Span ± SEM %)	EC50 (nM)<br>pEC50±SEM	Efficacy,<br>(Span ± SEM %)	EC50 (nM)<br>pEC50±SEM	Efficacy,<br>(Span ± SEM %)
WT	4	ND	ND	1.21<br>8.95±0.10	100.00±0.00	ND	ND

cAMP Accumulation

Construct	Replicates	DMSO	3 μM BMS-986187		10 μM BMS-986187	
MOR		EC50 (nM)<br>pEC50±SEM	EC50 (nM)<br>pEC50±SEM	Intrinsic Efficacy,<br>(Δspan ± SEM %)	EC50 (nM)<br>pEC50 ± SEM	Intrinsic Efficacy,<br>(Δspan ± SEM %)
WT	3	5.00<br>8.27±0.22	1.26<br>8.83±0.06	12.55±4.37	0.55<br>9.22±0.06	27.14±6.52
W194A	4	1.74<br>8.74±0.11	ND	70.26±4.41	ND	74.42±2.11
M205A	4	7.45<br>8.22±0.21	6.40<br>8.24±0.13	-1.86±5.07	3.99<br>8.40±0.10	1.50±8.40
Y151L	4	0.36<br>9.55±0.22	0.53<br>9.36±0.16	1.54±7.91	0.45<br>9.34±0.05	2.58±5.21
Y151A	3	0.17<br>9.85±0.16	0.24<br>9.77±0.36	9.65±7.65	0.26<br>9.68±0.22	5.47±4.83
Y151F	3	1.33<br>8.94±0.08	0.21<br>9.81±0.23	29.76±4.48	0.18<br>10.02±0.26	53.96±4.52
L118A	4	3.20<br>8.58±0.09	1.14<br>9.11±0.23	9.76±2.98	0.57<br>9.42±0.09	27.61±10.13
I144A	5	1.38<br>8.90±0.09	0.29<br>9.61±0.26	5.36±1.41	0.18<br>9.9±0.26	32.95±4.80
I148A	5	0.15<br>9.94±0.13	0.04<br>10.48±0.25	10.55±3.7	0.05<br>10.52±0.30	39.61±4.24
S198A	3	0.20<br>9.75±0.18	0.27<br>9.64±0.17	14.02±5.08	0.20<br>9.89±0.24	29.59±1.79
DOR		EC50 (nM)<br>pEC50±SEM	EC50 (nM)<br>pEC50±SEM	Intrinsic Efficacy,<br>(Δspan ± SEM %)	EC50 (nM)<br>pEC50 ± SEM	Intrinsic Efficacy,<br>(Δspan ± SEM %)
WT	2	0.50<br>9.24±0.01	ND	70±9.58	ND	76.43±11.17
Y130A	2	0.03<br>10.63±0.14	0.02<br>10.61±0.43	2.04±39.04	0.04<br>11.17±0.91	1.82±36.42
W173A	2	1.47<br>8.99±0.16	ND	93.17±1.64	ND	101.19±8.95
KOR		EC50 (nM)<br>pEC50±SEM	EC50 (nM)<br>pEC50±SEM	Intrinsic Efficacy,<br>(Δspan ± SEM %)	EC50 (nM)<br>pEC50 ± SEM	Intrinsic Efficacy,<br>(Δspan ± SEM %)
WT	2	2.65<br>8.58±0.09	0.10<br>10±0.09	31.13±7.24	0.07<br>10.16±0.30	52.22±10.48
Y139A	2	0.80<br>9.23±0.35	0.29<br>9.71±0.53	7.99±7.21	0.41<br>9.70±0.75	8.54±7.20
W183A	2	1.59<br>8.83±0.51	ND	61.73±0.18	ND	72.66±2.54

Gi1 BRET

MOR Construct	Replicates	DMSO	3 μM BMS-986187		10 μM BMS-986187	
		EC50 (nM)<br>pEC50±SEM	EC50 (nM)<br>pEC50±SEM	Intrinsic Efficacy,<br>(Δspan ± SEM %)	EC50 (nM)<br>pEC50±SEM	Intrinsic Efficacy,<br>(Δspan ± SEM %)
WT	3	16.26<br>7.84±0.25	4.20<br>8.24±0.28	3.26±2.96	1.39<br>8.85±0.15	5.70±4.63

β-arrestin2 Recruitment

MOR Construct	Replicates	DMSO	3 μM BMS-986187		10 μM BMS-986187	
		EC50 (nM)<br>pEC50±SEM	EC50 (nM)<br>pEC50±SEM	Intrinsic Efficacy,<br>(Δspan ± SEM %)	EC50 (nM)<br>pEC50±SEM	Intrinsic Efficacy,<br>(Δspan ± SEM %)
WT	3	710.60<br>6.13±0.09	145.70<br>6.84±0.06	1.22±5.69	50.85<br>7.33±0.16	18.47±12.83

Supplementary Table 3 | Composition of the MD simulation boxes.

	BMS986187-DAMGO-bound MOR-G_{i1}	DAMGO-bound MOR-G_{i1}
Simulation Box Dimensions (Å)	160x157x152	161x154x147
Total Number of Atoms	267,595	258,151
Water Molecules	54,865	52,402
Salt Concentration	0.15M	0.15M
Cholesterol	166	171
POPC	181	167
POPE	189	178
POPS	54	54
PIP2	38	37
PSM	51	58
GM3	35	36

Supplementary Table 4 | Normalized cell surface expression levels of MOR constructs determined by ELISA. Source data are provided as a Source Data file.

Construct	WT	W194A	Y151A	Y151L	Y151F	M205A	S198A	I148A	I144A	L118A
Mean ±SEM(%)	100.00 ±2.82	114.16 ±8.46	104.75 ±7.41	102.19 ±3.81	108.09 ±6.71	99.40 ±4.51	117.61 ±10.01	104.34 ±6.83	118.71 ±11.60	70.87 ±6.76
