# Supplementary material for: Structural and dynamic studies uncover a distinct allosteric modulatory site at the µ-opioid receptor
Source: Nat Commun. 2026 May 4;17:6000. doi: 10.1038/s41467-026-72633-z (PMC13347004; doi:10.1038/s41467-026-72633-z)
Supplement: Supplementary file 2 — Reporting Summary [file 41467_2026_72633_MOESM2_ESM.pdf]

nature portfolio

Corresponding author(s): Daniel Wacker

Last updated by author(s): Mar 12, 2026

Reporting Summary

Nature Portfolio wishes to improve the reproducibility of the work that we publish. This form provides structure for consistency and transparency in reporting. For further information on Nature Portfolio policies, see our Editorial Policies and the Editorial Policy Checklist.

Statistics

For all statistical analyses, confirm that the following items are present in the figure legend, table legend, main text, or Methods section.

n/a	Confirmed	
☐	☒	The exact sample size (*n*) for each experimental group/condition, given as a discrete number and unit of measurement
☐	☒	A statement on whether measurements were taken from distinct samples or whether the same sample was measured repeatedly
☐	☒	The statistical test(s) used AND whether they are one- or two-sided<br>*Only common tests should be described solely by name; describe more complex techniques in the Methods section.*
☒	☐	A description of all covariates tested
☒	☐	A description of any assumptions or corrections, such as tests of normality and adjustment for multiple comparisons
☐	☒	A full description of the statistical parameters including central tendency (e.g. means) or other basic estimates (e.g. regression coefficient) AND variation (e.g. standard deviation) or associated estimates of uncertainty (e.g. confidence intervals)
☐	☒	For null hypothesis testing, the test statistic (e.g. *F*, *t*, *r*) with confidence intervals, effect sizes, degrees of freedom and *P* value noted<br>*Give P values as exact values whenever suitable.*
☒	☐	For Bayesian analysis, information on the choice of priors and Markov chain Monte Carlo settings
☒	☐	For hierarchical and complex designs, identification of the appropriate level for tests and full reporting of outcomes
☒	☐	Estimates of effect sizes (e.g. Cohen's *d*, Pearson's *r*), indicating how they were calculated

Our web collection on statistics for biologists contains articles on many of the points above.

Software and code

Policy information about availability of computer code

Data collection	CryoEM data collection on Titan Krios electron microscope equipped with a K3 direct electron detector
Data analysis	CryoSPARC v4.1.2, MotionCor2, Ctffind4, WinCoot 0.9.8.1, Phenix v1.19.2, MolProbity, PyMol 2.5.4, UCSF Chimera v1.16, UCSF ChimeraX v1.2.5, GraphPad Prism8, MODELLER, CHARMM-GUI, Gromacs, RDKit, RTransferEntropy, igraph

For manuscripts utilizing custom algorithms or software that are central to the research but not yet described in published literature, software must be made available to editors and reviewers. We strongly encourage code deposition in a community repository (e.g. GitHub). See the Nature Portfolio guidelines for submitting code & software for further information.

Data

Policy information about availability of data

All manuscripts must include a data availability statement. This statement should provide the following information, where applicable:

- Accession codes, unique identifiers, or web links for publicly available datasets
- A description of any restrictions on data availability
- For clinical datasets or third party data, please ensure that the statement adheres to our policy

Electrostatic potential maps and structure coordinates have been deposited in the Electron Microscopy Data Bank (EMDB) and the Protein Data Bank (PDB). Structures of the DAMGO/MOR-Gαi1-Gβ1-Gγ2 complex have been deposited under EMD-71871/PDB-9PUD, and a receptor-only model for the same map has been deposited under PDB-10TM. Structures of the BMS986187/DAMGO/MOR-Gαi1-Gβ1-Gγ2 complex have been deposited under EMD-71869/PDB-9PU5 and a receptor-only model for the same map has been deposited under PDB-10TL.

Research involving human participants, their data, or biological material

Policy information about studies with human participants or human data. See also policy information about sex, gender (identity/presentation), and sexual orientation and race, ethnicity and racism.

Reporting on sex and gender	N/A
Reporting on race, ethnicity, or other socially relevant groupings	N/A
Population characteristics	N/A
Recruitment	N/A
Ethics oversight	N/A

Note that full information on the approval of the study protocol must also be provided in the manuscript.

Field-specific reporting

Please select the one below that is the best fit for your research. If you are not sure, read the appropriate sections before making your selection.

- [x] Life sciences
- [ ] Behavioural & social sciences
- [ ] Ecological, evolutionary & environmental sciences

For a reference copy of the document with all sections, see nature.com/documents/nr-reporting-summary-flat.pdf

Life sciences study design

All studies must disclose on these points even when the disclosure is negative.

Sample size	Sample size was not predetermined. Sample sizes were chosen based on years of experience with in vitro signaling assays, as well as based on sample sizes generally used and accepted in the field. For instance, for signaling studies we used a sample size of 2 or more independent experiments done in triplicates (technical repeats) to show reproducibility.
Data exclusions	No data was excluded
Replication	Experimental findings were reproduced, and number of technical repeats and independent experiments has been provided in the methods and figure legends.
Randomization	We typically observe low variability due to the use of experiments performed in neighboring wells of 384-well plates. Based on these results we did not perform randomization.
Blinding	No blinding was required for the experiments performed in this study, as all measures rely on instrument-based quantification of fluorescence and no subjective assessment of data that influences the outcome of the analysis.

Reporting for specific materials, systems and methods

We require information from authors about some types of materials, experimental systems and methods used in many studies. Here, indicate whether each material, system or method listed is relevant to your study. If you are not sure if a list item applies to your research, read the appropriate section before selecting a response.

Materials & experimental systems

n/a	Involved in the study	
	x	Antibodies
	x	Eukaryotic cell lines
x		Palaeontology and archaeology
x		Animals and other organisms
x		Clinical data
x		Dual use research of concern
x		Plants

Methods

n/a	Involved in the study	
x		ChIP-seq
x		Flow cytometry
x		MRI-based neuroimaging

Antibodies

Antibodies used	anti-flag FITC antibody (Genscript, A01632)
Validation	This antibody was used in experiments comparing its activity in transfected vs untransfected cells using a 1:500 dilution

Eukaryotic cell lines

Policy information about cell lines and Sex and Gender in Research

Cell line source(s)	Sf9 cells (Expression Systems, 94-001S), HEK293T Cell Line (ATCC, CRL-3216)
Authentication	None of the cell lines used was authenticated
Mycoplasma contamination	All cell lines used in this study tested negative for mycoplasma contamination
Commonly misidentified lines (See ICLAC register)	No commonly misidentified cell lines were used

Plants

Seed stocks	N/A
Novel plant genotypes	N/A
Authentication	N/A
