# Supplementary material for: Structural and dynamic studies uncover a distinct allosteric modulatory site at the µ-opioid receptor
Source: Nat Commun. 2026 May 4;17:6000. doi: 10.1038/s41467-026-72633-z (PMC13347004; doi:10.1038/s41467-026-72633-z)
Supplement: Supplementary file 3 — Transparent Peer Review file [file 41467_2026_72633_MOESM3_ESM.pdf]

nature portfolio

Peer Review File

Structural and dynamic studies uncover a distinct allosteric modulatory site at the μ-opioid receptor

Corresponding Author: Dr Daniel Wacker

This file contains all reviewer reports in order by version, followed by all author rebuttals in order by version.

Version 0:

Reviewer comments:

Reviewer #1

(Remarks to the Author)
Comments on NCOMMS-25-65960
In this manuscript the authors reported a known positive allosteric modulator of the DOR bound to the MOR at a lipid-receptor interface binding site. Overall the quality of the work is sound. The data analysis seems reasonable. The discussion of the results is on the conservative side. The information provided by the report is new in the field while there is limited new knowledge contributed to the research community. As the authors agreed, it is far-fetching to benefit therapeutic development targeting the MOR.

Reviewer #2

(Remarks to the Author)
The manuscript by Zhang and colleagues investigates the molecular basis of μ opioid receptor regulation by the PAM BMS-986187 using cryo-EM, MD simulations, and signalling assays combined with receptor mutagenesis. They uncover that BMS-986187 binds MOR in a binding pocket that is distinct from previously described allosteric sites of another PAM and NAM. They then propose a model for allosteric communication between BMS-986187 binding and the DAMGO-occupied orthosteric site and the G protein interface. The results are interesting and novel, the mechanistic insights into BMS-986187 binding allow a deepened understanding of MOR regulation, which can be instructive for future therapeutic developments.
However, several points need to be addressed:
1) The authors resolve the structure of the MOR-G protein complex bound or not by BMS-986187 in an antiparallel dimer arrangement (which is non-physiological / formed post-lysis). The authors need to include a figure that depicts the details of the MOR dimer complex to clarify if BMS-986187 binding may be affected by the non-physiological receptor dimer.
2) It is unclear why the signalling assays (cAMP and BRET-based G protein recruitment) involve a 30 min pre-incubation step with DAMGO or BMS-986187. It is well established that desensitization of MOR occurs within minutes of agonist addition, which complicates the interpretation of the presented data. Since G protein coupling (BRET readout) and G protein-medicated inhibition of ACs (cAMP readout) happen rapidly upon MOR activation, the cAMP and BRET data should have been recorded immediately following agonist addition. The authors need to test if the 30 min pre-incubation window affects their signalling data. In the context of MOR regulation, it would also be important to probe if beta-arrestin binding to MOR is influenced by BMS-986187, similar to G protein signalling.
3) To test MOR residues involved in BMS-986187 binding, the authors test a series of MOR mutants regarding their effects on the concentration-dependent DAMGO response in the presence of BMS-986187 or the PAM BMS-986187, with the response normalized between 0-100% for each receptor mutant. Since the MOR mutations lie in the transmembrane domains and therefore likely impair receptor folding or trafficking, it would be important to show the surface levels of the MOR mutants as well as their DAMGO affinity, to understand the validity of the data & interpretation.
4) The analyses of information flux between the PAM, the orthosteric binding site, and the G protein interface is interesting. Experimental data that support the proposed central role of the cholesterol molecule close to the BMS-986187 binding site are needed to support the findings. How does acute modulation of cholesterol levels (e.g. using methyl-beta-cyclodextrin or exogenous cholesterol addition) affect BMS-986187's regulation of MOR signalling?
5) BMS-986187 was first discovered as a PAM of the delta opioid receptor, and acts as a PAM of the kappa opioid receptor, too. This is discussed by the authors, yet it would have been important to perform a mutational analysis of the conserved residues in DOR and KOR, to test if the BMS-986187 binding site is comparable between opioid receptor types or unique to

MOR.

Reviewer #3

(Remarks to the Author)
This is an interesting study, and the topic on distinct allosteric modulatory site at the μ-opioid receptor and positive allosteric modulators with therapeutic potential is timely. Generally, the work appears to be satisfactorily described and the data are correctly analyzed.

There are some points to be addressed by the authors.

The authors should better rationalize the choice of BMS-986187 as PAM MOR for their study. BMS-986187 is reported as a dual DOR and KOR PAM, being selective for these receptors over the MOR with an approximately 20- to 30- fold higher affinity in the allosteric ternary complex mode (doi:10.1124/mol.117.109561). BMS-986187 is a derivative of the BMS-986122, a MOR PAM, therefore, it would be important to include the structure of BMS-986122. Showing the structures of other PMAP mentioned in the Introduction in a Supporting Figure, it would be valuable for the readers.

In the Introduction and/or Discussion, I recommended a useful reference to the side effects of opioids doi: 10.3390/ph14111091.

Line 104 – A rather high dose of 100 μM BMS-986187 was used. Why?

Who provided BMS-986187? How was BMS-986187 solved for the in vitro functional assays? What is exactly "DMSO" as vehicle? Which final concentration of DMSO was tested? Suggest changing "DMSO" to vehicle in Figures and Tables for better reading.

Line 495 – Is it 27oC or 37oC?

Extended Table 2 – change uM to μM, define NS.

Reviewer #4

(Remarks to the Author)
Reviewed article: "Structural and dynamic studies uncover a distinct allosteric modulatory site at the μ-opioid receptor."

Overall recommendation: accept after minor revisions noted.

Summary: In this study, the authors report a cryo-EM structure of the μ-opioid receptor (MOR) in complex with BMS-986187, a positive allosteric modulator (PAM) of opioid receptors (ORs). Although this PAM was previously shown to interact with other ORs, the resolved structure reveals a novel allosteric binding site specific to MOR. This finding has significant pharmacological implications and is of broad interest to the GPCR community, as identifying new modulator binding sites expands the potential for drug discovery targeting MOR. The authors strengthen this key observation with both computational and experimental evidence. Molecular dynamics simulations of two cryo-EM structures, combined with contact frequency analysis, were used to identify residues for mutational studies. The resulting experiments confirmed that these interactions are critical for allosteric regulation. Notably, while alanine substitution of several residues abolished allosteric modulation, the W194A mutation enhanced it, producing nearly identical dose-response curves at 3 and 10 μM and suggesting new directions for studying MOR activity regulation. The authors further applied an information-theoretic analysis, quantifying effective transfer entropy between selected residues to establish causal relationships between the allosteric site, the orthosteric site, and the G-protein interface.

Comments:

This review is focused on the MD simulations and theoretical aspects of the study.

Overall, the authors employ standard and community-accepted protocols to carry out their simulations. This includes using state-of-the-art force fields, complex membrane composition, and reasonable equilibration/burn-in times. The time length of the independent production runs is quite short (500 ns), while processes such as ligand binding in proteins like GPCRs typically take hundreds of μs or more. Nonetheless, since the goal of the authors is to observe thermodynamic fluctuations within the DAMGO-bound, PAM-bound metastable state to measure contact frequencies, the sampling time and number of independent replicates is enough to obtain converged statistics. The fact that the residues selected based on the MD analysis had a clear impact during the mutational experiments further lends credibility to the computational predictions. Regarding the effective transfer entropy calculation, this complementary statistical analysis allows the authors to make an additional claim about the causality of the allosteric effect, that is, that the allosteric site influences other regions of the protein but not vice versa. However, the manuscript does not specify the order of the Markov process used to model the discrete, time-dependent variables. Both the referenced analysis method (ref. 46) and software implementation (ref. 76) emphasize the importance of this parameter. Based on the formula shown on line 704, it appears that a first-order process was used, and the delay parameter (τ) was set to match the simulation save frame rate of 1 ns. From my experience with protein systems, it is unlikely that the conformational states of these residues become history-independent on such a short

timescale.
I suggest two possible ways to strengthen this analysis:
(1) Estimate an appropriate τ value by computing the time autocorrelations of the discrete variables and selecting a delay such that the autocorrelation of the slowest variable has decayed beyond a threshold (e.g., 95%). While the decorrelation time may exceed 1 ns, this approach would provide empirical justification for your choice. It also allows you to maintain a first-order Markov model (lx = ly = 1 in the software package). You can then re-plot the results to verify whether the conclusions remain consistent.

(2) Vary the Markov orders (lx and ly) systematically, for instance, setting lx = ly = 1, 2, 3, etc., until computational limits or statistical noise become prohibitive (keeping lx = ly, because your variables are of the same kind). Then compare the results across these parameter settings to assess the robustness of your conclusions.

That said, I recognize that the MD simulations were primarily performed for the contact frequency analysis and that the core findings of the paper are not contingent on the outcome of the transfer entropy analysis. For this reason, I do not consider this issue critical enough to delay publication. If the additional analysis supports your current conclusions, a brief note to that effect would suffice. If, however, the results turn out to be sensitive to parameter choice, the authors should clarify that the transfer entropy results 'suggest' a causal relationship in allosteric modulation, but that further evidence, such as longer trajectories, would be needed to fully rule out memory-dependent effects in MOR dynamics.

Version 1:

Reviewer comments:

Reviewer #2

(Remarks to the Author)
The authors have addressed all my comments and concerns by adding a significant amount of new data and discussion points. I support the publication of the revised manuscript.

Reviewer #3

(Remarks to the Author)
The authors have satisfactorily addressed all my concerns.

Reviewer #4

(Remarks to the Author)
This review addresses the revised version of the manuscript entitled “Structural and dynamic studies uncover a distinct allosteric modulatory site at the µ-opioid receptor.”

The authors have satisfactorily addressed the previous concern regarding parameter selection for the transfer entropy calculations. While varying the Markov model order leads to changes in a subset of the top 10% interacting pairs, the high Spearman correlation indicates that the overall rankings are largely preserved.

I therefore support publication of the manuscript in its current form.

Reviewer #5

(Remarks to the Author)
In their manuscript entitled “Structural and dynamic studies uncover a distinct allosteric modulatory site at the µ-opioid receptor”, Zhang et al. present high resolution structures of the µ-opioid receptor (MOR) bound to the orthosteric agonist DAMGO with and without a cobound positive allosteric modulator (PAM), BMS-986187. Whilst BMS-986187 is a well known and well studied PAM of the opioid receptor family, it’s binding site has remained unknown. The structure presented here reveals BMS-986187 binds to a novel allosteric site at the opioid receptor family. Using a combination of mutagenesis, signalling assays combined with molecular dynamics simulations and analysis, the authors provide mechanistic insights into how BMS-986187 allosterically modulates MOR function through this allosteric binding site. The findings are novel to the MOR and wider opioid receptor family and have the potential to aid in the design of novel allosteric modulators for this important therapeutic target.
To improve the manuscript, the following points should be addressed.
Main Points
1. My main concern regarding the Cryo-EM maps is the poor density for the G proteins. One explanation for this is that there is partial occupancy of G protein within the dimer (perhaps due to a clash of the G protein with the extracellular loops of MOR) such that only one of the MOR protomers is bound to G protein, and averaging of this occupancy across the two protomers leads to poor density for the G protein. As the authors state, the anti-parallel dimer species was also observed in

previous Opioid Receptor-G protein structures (PMID: 36368306, 36368306). I note that the authors in these manuscripts implemented processing strategies that likely helped in producing high-resolution maps for the entire receptor-G protein complex, strategies which I deduce were not implemented here. For example, PMID: 36368306 performed local refinement with a mask around one G protein, whereas in this manuscript only masks around receptors (monomer vs dimer) were implemented. I recommend trying a similar processing strategies as PMID: 36368306, 36368306 to see if this will improve map quality. Another strategy that may help is to symmetry expand particles using C2 symmetry and then performing 3D classification with the appropriate masks.
Obtaining higher quality density for the G protein will allow the authors to confidently model G protein and allow for expanded discussion on a number of items, including the observed shifts in $G\alpha_{i1}$ (line 222) and the PAM induced differences at the MOR-Gαi1 interface (line 244-246).

Without improved density for the G protein, the authors should reconsider depositing models of the entire receptor–G protein complex. Whilst I commend the authors for omitting the AHD domain of the Gα protein due to insufficient density, the density for the RAS domain is also poor and limited. Without improved density, there is currently insufficient data to support modelling of the entire G protein.

2. It appears DAMGO is slightly mismodelled with the “F5” moiety of DAMGO positioned outside of what appears reasonable density for this part of the molecule. This is most apparent in the BMS-986187_DAMGO bound structure with the deepEMhancer map (figure uploaded as part of the review). It is less apparent in the DAMGO only structure which is likely due to poorer density for this part of the molecule in the DAMGO only maps. The authors should carefully consider remodelling DAMGO. If ultimately the pose differs between the BMS-986187 bound and unbound structures, a discussion on whether there is an allosteric influence of BMS-986187 on DAMGO's pose is warranted.

3. Beginning line 331, the manuscript contains a sizeable discussion on the rotameric states of $Y151^{3.34}$ and $W194^{4.50}$ during the MD simulations of PAM bound and unbound MOR complexes. Yet the manuscript lacks supporting data to visualise these rotameric transitions. To substantiate the claims made in this section, I recommend including figure(s) that directly illustrate the rotameric states of these residues throughout the simulations.

4. A major finding of this manuscript is the 1) central role of $W194^{4.50}$ in facilitating allosteric transitions at the MOR and the wider opioid receptor family and it’s ability to enhance the allosteric effects of PAM’s, and 2) the role of cholesterol and the influence that $W194^{4.50}$ has on cholesterol and the potential interplay between PAM+cholesterol+$W194^{4.50}$. There are a few points relating to this that are not adequately covered in the manuscript.
• $W^{4.50}$ is one of the most highly conserved residues in class A GPCRs and its role in facilitating allosteric modulation has been investigated at other GPCRs including the CB1 (PMID: 31659318, 35637350).
• The cholesterol site discovered for the MOR is a well known and well characterised cholesterol binding site at class A GPCRs (PMID: 18547522, 18547522)
A discussion on how the findings at the MOR compare and contrast to these established findings would improve the manuscript.

5. In relation the the above point, another focus of this manuscript is the differences between BMS-986187 across the MOR, DOR and KOR, and the potential differences in the role for cholesterol in mediating this effect (section beginning line 435). The current discussion of cholesterol's role in this context remains limited and would benefit substantially from newly available structural data. Notably, density for cholesterol molecules has now been observed in the same site at the DOR bound to BMS-986187 and derivatives (recent preprint https://doi.org/10.1101/2025.10.16.682975), contradicting the statement at line 444 that available DOR structures lack a cholesterol molecule at the corresponding site. With these structures now available in the PDB and EMDB, a comprehensive comparative analysis of cholesterol positioning and occupancy with the DOR is now feasible. Such an analysis would substantially strengthen the current discussion—which tentatively suggests that "BMS-986187 may exert subtly distinct modulatory effects at DOR, a possibility that warrants further investigation" (line 441)—by providing direct structural evidence to either support or refine conclusions regarding PAM selectivity and mechanism.

Minor Points
1. There is an inconsistency in the reported concentration of BMS-986187 during the purification process. The results section (line 107) states that 100 μM was used, whilst the methods section (line 540) specifies 30 μM. Please clarify/rectify.

2. Line 297-298. “Unexpectedly, a $W194^{4.50}$A mutation significantly enhanced the allosteric effects of BMS-986187, both by increasing the PAM’s intrinsic efficacy (Fig 4D) and by lowering the concentration required to achieve this effect (Fig. 4A).”
This is two sides of the same coin. If the intrinsic efficacy of BMS-986187 is enhanced at this mutant, then it is expected a reduced concentration of BMS-986187 leads to efficacy.

3. Line 647 states a concentration of 1 nM was used to stimulate cAMP production. This concentation seems rather low. MedChemExpress (the stated supplier) lists an EC50 value of 500 nM of Foskolin for Adenylyl Cyclase. A 1 nM concentration would fall well below the threshold required to produce the suitable increase in cAMP required for observing inhibition via Gi1 activation. Concentrations of 10-100 μM forskolin are routinely used in these assays.

4. In the figure legends for Figure 1A and Figure 2A, please mention the ligands are shown as sticks and transparent spheres to avoid confusing the transparent spheres for ligand density.

5. For extended figure 2D, can the authors include which map was used to represent the density?

Version 2:

Reviewer comments:

Reviewer #5

(Remarks to the Author)
The authors have addressed all of my points. I thank them for taking a considered and measured approach. The manuscript accurately represents their findings and in my opinion, is ready for publication.

Open Access This Peer Review File is licensed under a Creative Commons Attribution 4.0 International License, which permits use, sharing, adaptation, distribution and reproduction in any medium or format, as long as you give appropriate credit to the original author(s) and the source, provide a link to the Creative Commons license, and indicate if changes were made.
In cases where reviewers are anonymous, credit should be given to 'Anonymous Referee' and the source.
The images or other third party material in this Peer Review File are included in the article's Creative Commons license, unless indicated otherwise in a credit line to the material. If material is not included in the article's Creative Commons license and your intended use is not permitted by statutory regulation or exceeds the permitted use, you will need to obtain permission directly from the copyright holder.
To view a copy of this license, visit https://creativecommons.org/licenses/by/4.0/

REVIEWER COMMENTS

We thank the reviewers for their kind comments and constructive feedback on our work. We have carefully evaluated all comments and addressed them below, which included numerous additional experiments that have either been included in the manuscript, or, that show that much more work is required.

We would also like to point out that while performing experiments for the revision, our experiments revealed intrinsic agonist activity for BMS-986187 at MOR in our cAMP assays, which was previously not observed (former Figure 1B in the initial submission). These new data have now been included in the revised manuscript (Figure 1C) and agree with findings in the literature (PMID: 29233847) that we had already previously cited. It should be noted that **this does not affect the overall findings or conclusion of our study**, but explains the BMS-986187-mediated increase in efficacy at low DAMGO concentrations. We have now revised the manuscript to remove mentioning of modulating “base efficacy” and “Emin”, and rather attribute this effect to the intrinsic efficacy of BMS-986187 throughout the manuscript.

Reviewer #1 (Remarks to the Author):

Comments on NCOMMS-25-65960

In this manuscript the authors reported a known positive allosteric modulator of the DOR bound to the MOR at a lipid-receptor interface binding site. Overall the quality of the work is sound. The data analysis seems reasonable. The discussion of the results is on the conservative side. The information provided by the report is new in the field while there is limited new knowledge contributed to the research community. As the authors agreed, it is far-fetching to benefit therapeutic development targeting the MOR.

We would like to thank the reviewer for the kind words on our rigor, impact, and mindful discussion of our findings in a broader therapeutic context.

Reviewer #2 (Remarks to the Author):

The manuscript by Zhang and colleagues investigates the molecular basis of μ opioid receptor regulation by the PAM BMS-986187 using cryo-EM, MD simulations, and signalling assays combined with receptor mutagenesis. They uncover that BMS-986187 binds MOR in a binding pocket that is distinct from previously described allosteric sites of another PAM and NAM. They then propose a model for allosteric communication between BMS-986187 binding and the DAMGO-occupied orthosteric site and the G protein interface. The results are interesting and novel, the mechanistic insights into BMS-986187 binding allow a deepened understanding of MOR regulation, which can be instructive for future therapeutic developments.

However, several points need to be addressed:

1) The authors resolve the structure of the MOR-G protein complex bound or not by BMS-986187 in an antiparallel dimer arrangement (which is non-physiological / formed post-lysis). The authors need to include a figure that depicts the details of the MOR dimer complex to clarify if BMS-986187 binding may be affected by the non-physiological receptor dimer.

We thank the reviewer for this suggestion. We have now added an extended figure illustrating the MOR dimer arrangement in our structures. As shown in this figure (Figure A), BMS-986187 is positioned on the outer surface of the receptor and is spatially separated from the dimer interface. Notably, a similar binding site for BMS-986187 has been reported for the δ-opioid receptor in a bioRxiv preprint (https://doi.org/10.1101/2025.10.16.682975), supporting the notion that this site is conserved and likely independent of dimer formation. Based on the available structural information, it therefore appears unlikely that the non-physiological dimer arrangement observed in our structures significantly influences BMS-986187 binding. We have now included this figure in the manuscript (Extended Data Fig. 2A), and stated that the dimerization likely does not influence PAM binding (Line 124-126, 213-214).

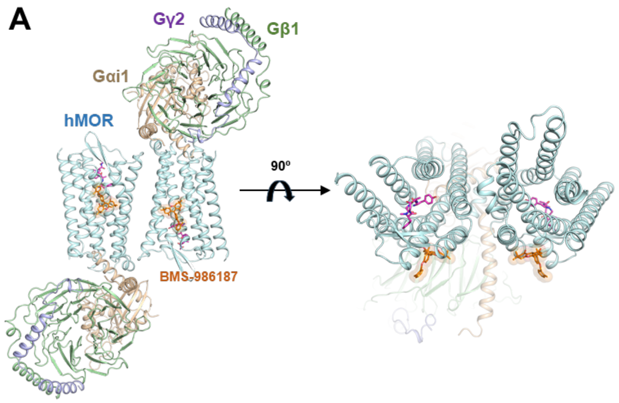

2) It is unclear why the signalling assays (cAMP and BRET-based G protein recruitment) involve a 30 min pre-incubation step with DAMGO or BMS-986187. It is well established that desensitization of MOR occurs within minutes of agonist addition, which complicates the interpretation of the presented data. Since G protein coupling (BRET readout) and G protein-medicated inhibition of ACs (cAMP readout) happen rapidly upon MOR activation, the cAMP and BRET data should have been recorded immediately following agonist addition. The authors need to test if the 30 min pre-incubation window affects their signalling data. In the context of MOR regulation, it would also be important to probe if beta-arrestin binding to MOR is influenced by BMS-986187, similar to G protein signalling.

This is indeed an important point, and we acknowledge that MOR desensitization can occur within minutes, as is the case for many GPCRs. However, measuring MOR desensitization in real time is not practical for the objectives of our study, which was aimed at comparing the effects of multiple drugs, drug concentrations, and receptor mutations. Such comparisons require recording many conditions in parallel, and our 384-well plate readout requires approximately 15 minutes for both the BRET and cAMP measurements; therefore, a pre-incubation period was used to ensure uniform exposure times across all wells during plate reading.

Nevertheless, the reviewer's point is well taken, and we have evaluated pre-incubation times ranging from 15 to 45 minutes (Figure B). The resulting data showed no obvious qualitative differences attributable to incubation time, although minor variations in absolute readouts were observed, as expected. While it remains possible that incubation times shorter than 15 min could uncover PAM- or mutation-mediated effects on MOR desensitization, we believe that such effects do not affect the findings or conclusions of our current study. We have nonetheless included a sentence in the discussion explicitly noting this limitation and the potential role of MOR desensitization not captured in our assays (Line 459-461).

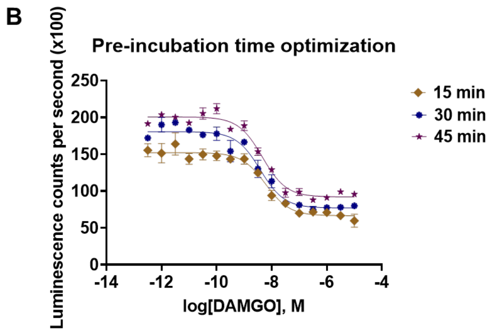

In addition, we appreciate the suggestion regarding β-arrestin, and have since performed β-arrestin2 recruitment assays in the presence and absence of BMS-986187 (Figure C). We find that indeed, BMS-986187 also allosterically increases DAMGO's ability to recruit β-arrestin2 to MOR. We have now included this new result in the revised manuscript (Fig. 1B).

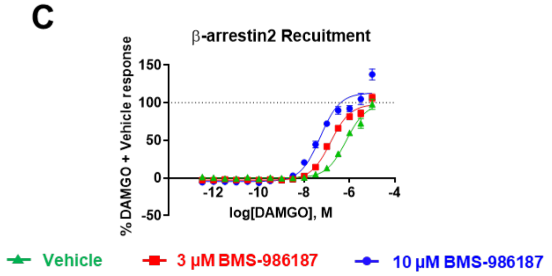

3) To test MOR residues involved in BMS-986187 binding, the authors test a series of MOR mutants regarding their effects on the concentration-dependent DAMGO response in the presence of BMS-986187 or the PAM BMS-986187, with the response normalized between 0-100% for each receptor mutant. Since the MOR mutations lie in the transmembrane domains and therefore likely impair receptor folding or trafficking, it would be important to show the surface levels of the MOR mutants as well as their DAMGO affinity, to understand the validity of the data & interpretation.

We agree that assessing the surface expression levels of the MOR mutants is important for proper interpretation of the functional data. Accordingly, we performed cell-surface ELISA, and found that most of the mutations did not significantly alter MOR surface expression (Figure D). The only exception was L118A, which showed statistically significant reduction in cell-surface expression relative to WT. That being said, this residue does not appear to be directly involved in allosteric modulation by BMS-986187, as the L118A mutant shows no meaningful change in PAM-mediated enhancement of cAMP inhibition

compared with WT (Figure 4B, Extended Data Figure 5, Extended Data Table 2). Thus, the lower surface expression of L118A does not appear to affect receptor activity, which remains comparable to that of the wild-type receptor. We have now included the ELISA data in the revised manuscript as Extended Fig. 5B.

D

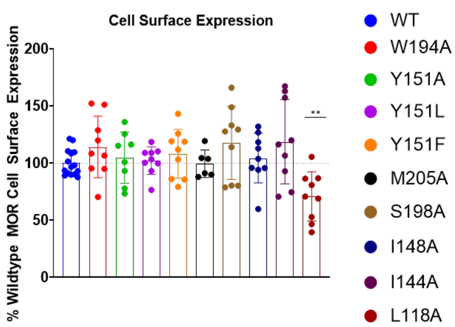

In contrast, determining DAMGO binding affinities for MOR and its mutants represents a substantial undertaking. In the context of the present study, establishing a single "correct" DAMGO affinity is itself non-trivial. Our primary interest lies in how BMS-986187 allosterically modulates DAMGO activity rather than in absolute ligand affinities. A rigorous affinity-based analysis would require measuring DAMGO binding to both inactive and active states of MOR and each mutant, since PAM effects are expected to influence the inactive-to-active state transition. This would likely necessitate reconstitution in nanodiscs and the addition of large amounts of G protein to stabilize the active state. We therefore feel that such extensive experiments fall beyond the scope of the current study and would not provide commensurate additional insight relative to the substantial effort required.

4) The analyses of information flux between the PAM, the orthosteric binding site, and the G protein interface is interesting. Experimental data that support the proposed central role of the cholesterol molecule close to the BMS-986187 binding site are needed to support the findings. How does acute modulation of cholesterol levels (e.g. using methyl-beta-cyclodextrin or exogenous cholesterol addition) affect BMS-986187's regulation of MOR signalling?

This is an excellent comment, as we share the reviewer's interest in the potential role of cholesterol near the BMS-986187 binding site. In response, we conducted a series of experiments aimed at providing initial insight into whether cholesterol depletion affects the ability of BMS-986187 to modulate MOR activity. However, these studies indicate that a substantially more comprehensive investigation would be required to reach definitive conclusions, and such an effort would extend beyond the scope of a revision.

Our first goal was to identify experimental conditions under which cholesterol depletion produces a measurable global effect on MOR signaling while still preserving a sufficient dynamic range to assess PAM-mediated effects in our cAMP assay. We first optimized incubation times and media/buffer conditions for cholesterol depletion. Next, we tested various methyl-beta-cyclodextrin (MβCD) concentrations and found that when cells were treated with 1 mM MβCD for 30 min in DMEM, we saw a substantial effect on the DAMGO concentration response (Figure E). Concentrations including and above 2 mM, however, severely compromised the signal window and precluded reliable assessment of PAM effects, and less than 1 mM MβCD had only negligible effects. We thus measured BMS-986187's effects on DAMGO signaling under normal conditions and following cholesterol depletion using 1 mM MβCD in our cAMP inhibition assay. Analysis of the data shows that cholesterol depletion using a concentration of 1 mM MβCD did not statistically alter the PAM-induced effects on MOR signaling (Figure E). This could

be explained by several, non-mutually exclusive possibilities: 1) 1 mM MβCD may not remove sufficient amounts of the cholesterol molecule proximal to the PAM site to impact PAM activity at MOR; 2) broader perturbations arising from cholesterol depletion at MOR or elsewhere in the cell may mask effects specifically attributable to disruption of a PAM–cholesterol interaction; or 3) the nearby CLR molecule may not play a functionally significant role in BMS-986187's PAM activity. Taken together, our observations suggest that more extensive experimental work (including increasing cholesterol concentrations) will be required to address this question, which we plan to pursue in future studies.

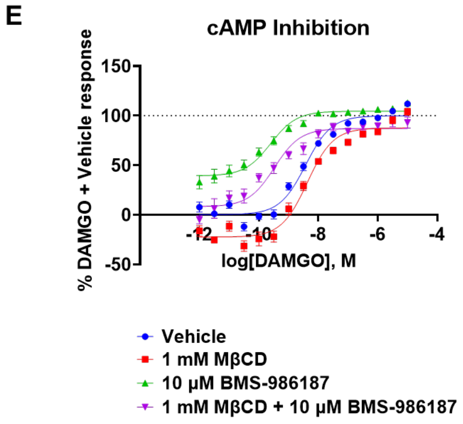

5) BMS-986187 was first discovered as a PAM of the delta opioid receptor, and acts as a PAM of the kappa opioid receptor, too. This is discussed by the authors, yet it would have been important to perform a mutational analysis of the conserved residues in DOR and KOR, to test if the BMS-986187 binding site is comparable between opioid receptor types or unique to MOR.

Previous studies have indeed reported that BMS-986187 exhibits selectivity for DOR, and we agree with the reviewer that this observation should be validated in our assay system. Accordingly, we performed the same cAMP assay used for MOR to evaluate PAM activity at DOR and KOR. In these experiments, BMS-986187 modulated DADLE-mediated signaling at DOR and U-69593-mediated signaling at KOR (Figure F). At 3 μM concentration, BMS-986187 displayed substantially stronger PAM activity at DOR than at MOR or KOR, thereby confirming its preference for DOR and validating prior reports. We further tested the effects of mutating W4.50 and Y3.34, residues that form part of the allosteric pocket in MOR, on PAM activity at DOR and KOR. These mutations produced trends similar to those observed for MOR. Specifically, the Y3.34A mutations strongly reduced PAM-mediated effects at both DOR and KOR, whereas the W4.50A mutation increased BMS-986187's activity at KOR and DOR. Collectively, these results indicate that the BMS-986187 binding site is comparable between MOR, KOR, and DOR. This is further supported by a bioRxiv preprint (https://doi.org/10.1101/2025.10.16.682975), suggesting that a BMS-986187 analog binds to a similar binding site at DOR. Collectively, the new data on DOR and KOR further support our mechanistic model in which W4.50 and Y3.34 play critical roles in regulating the inactive-to-active state transition of opioid receptors and, consequently, in enabling positive allosteric modulation by BMS-986187. These data have now been included in the revised manuscript in Extended Fig 6C.

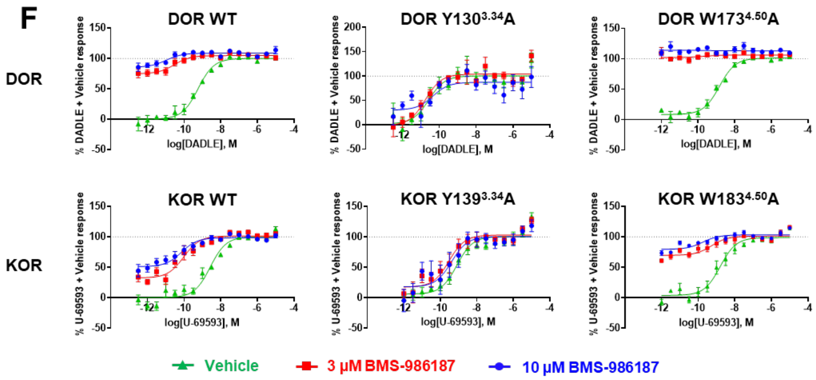

Reviewer #3 (Remarks to the Author):

This is an interesting study, and the topic on distinct allosteric modulatory site at the μ-opioid receptor and positive allosteric modulators with therapeutic potential is timely. Generally, the work appears to be satisfactorily described and the data are correctly analyzed.

There are some points to be addressed by the authors.

The authors should better rationalize the choice of BMS-986187 as PAM MOR for their study. BMS-986187 is reported as a dual DOR and KOR PAM, being selective for these receptors over the MOR with an approximately 20- to 30- fold higher affinity in the allosteric ternary complex mode (doi:10.1124/mol.117.109561). BMS-986187 is a derivative of the BMS-986122, a MOR PAM, therefore, it would be important to include the structure of BMS-986122. Showing the structures of other PMAP mentioned in the Introduction in a Supporting Figure, it would be valuable for the readers.

We thank the reviewer for this great suggestion and have now included the chemical structures of relevant opioid PAMs (Figure G) in Figure 2B. The central premise of our study was to elucidate modulatory surfaces at MOR by examining the mechanism of action of a PAM that is structurally and thus likely pharmacologically distinct from BMS-986122. While we agree (and now show in Extended Data Fig 6C) that BMS-986187 exhibits higher selectivity for DOR, we use BMS-986187 here as a tool to investigate MOR allosteric modulation and to enable a structural comparison with BMS-986122. We have accordingly made minor revisions to the introduction to more clearly articulate this rationale.

G

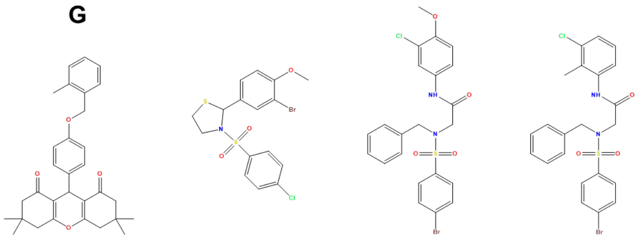

In the Introduction and/or Discussion, I recommended a useful reference to the side effects of opioids doi: 10.3390/ph14111091.

We thank the reviewer for this suggestion and have now included this citation in the introduction (Line 46).

Line 104 – A rather high dose of 100 μM BMS-986187 was used. Why?

For structural studies, we routinely employ high compound concentrations as ligands often exhibit substantially reduced affinities for purified receptors compared with their membrane-embedded

counterparts. Accordingly, we used a concentration of 100 µM during the final steps to ensure high receptor occupancy, thereby facilitating reliable structural elucidation of the bound compound.

Who provided BMS-986187? How was BMS-986187 solved for the in vitro functional assays? What is exactly “DMSO” as vehicle? Which final concentration of DMSO was tested? Suggest changing “DMSO” to vehicle in Figures and Tables for better reading.

We apologize for the omission and have now updated the methods section accordingly. BMS-986187 was purchased from MedChemExpress and dissolved in DMSO to prepare a 10 mM stock solution. Working solutions were prepared at 3x the final concentration and added to cells in our final buffer (1x HBSS + 0.01% BSA + 0.01% Ascorbic Acid). DMSO was used as the vehicle control and diluted in an identical manner, such that all vehicle and compound conditions contained the same exact DMSO concentration. To improve clarity and facilitate interpretation of the data, we have explicitly stated the use of DMSO as the vehicle control in the revised manuscript and have replaced “DMSO” with “Vehicle” in all corresponding data graphs.

Line 495 – Is it 27oC or 37oC?

It is indeed 27 °C as previously indicated. This is the optimal temperature of culturing Sf9 insect cells used for producing protein for structural studies.

Extended Table 2 – change uM to µM, define NS.

We have now addressed the unit typo and defined ND as “Not determined” in the legend.

Reviewer #4 (Remarks to the Author):

Reviewed article: "Structural and dynamic studies uncover a distinct allosteric modulatory site at the μ-opioid receptor."

Overall recommendation: accept after minor revisions noted.

Summary: In this study, the authors report a cryo-EM structure of the μ-opioid receptor (MOR) in complex with BMS-986187, a positive allosteric modulator (PAM) of opioid receptors (ORs). Although this PAM was previously shown to interact with other ORs, the resolved structure reveals a novel allosteric binding site specific to MOR. This finding has significant pharmacological implications and is of broad interest to the GPCR community, as identifying new modulator binding sites expands the potential for drug discovery targeting MOR. The authors strengthen this key observation with both computational and experimental evidence. Molecular dynamics simulations of two cryo-EM structures, combined with contact frequency analysis, were used to identify residues for mutational studies. The resulting experiments confirmed that these interactions are critical for allosteric regulation. Notably, while alanine substitution of several residues abolished allosteric modulation, the W194A mutation enhanced it, producing nearly identical dose-response curves at 3 and 10 μM and suggesting new directions for studying MOR activity regulation. The authors further applied an information-theoretic analysis, quantifying effective transfer entropy between selected residues to establish causal relationships between the allosteric site, the orthosteric site, and the G-protein interface.

Comments:

This review is focused on the MD simulations and theoretical aspects of the study.

Overall, the authors employ standard and community-accepted protocols to carry out their simulations. This includes using state-of-the-art force fields, complex membrane composition, and reasonable equilibration/burn-in times. The time length of the independent production runs is quite short (500 ns), while processes such as ligand binding in proteins like GPCRs typically take hundreds of μs or more. Nonetheless, since the goal of the authors is to observe thermodynamic fluctuations within the DAMGO-bound, PAM-bound metastable state to measure contact frequencies, the sampling time and number of independent replicates is enough to obtain converged statistics. The fact that the residues selected based on the MD analysis had a clear impact during the mutational experiments further lends credibility to the computational predictions.

Regarding the effective transfer entropy calculation, this complementary statistical analysis allows the authors to make an additional claim about the causality of the allosteric effect, that is, that the allosteric

site influences other regions of the protein but not vice versa. However, the manuscript does not specify the order of the Markov process used to model the discrete, time-dependent variables. Both the referenced analysis method (ref. 46) and software implementation (ref. 76) emphasize the importance of this parameter. Based on the formula shown on line 704, it appears that a first-order process was used, and the delay parameter (τ) was set to match the simulation save frame rate of 1 ns. From my experience with protein systems, it is unlikely that the conformational states of these residues become history-independent on such a short timescale.

I suggest two possible ways to strengthen this analysis:

(1) Estimate an appropriate τ value by computing the time autocorrelations of the discrete variables and selecting a delay such that the autocorrelation of the slowest variable has decayed beyond a threshold (e.g., 95%). While the decorrelation time may exceed 1 ns, this approach would provide empirical justification for your choice. It also allows you to maintain a first-order Markov model (lx = ly = 1 in the software package). You can then re-plot the results to verify whether the conclusions remain consistent.

(2) Vary the Markov orders (lx and ly) systematically, for instance, setting lx = ly = 1, 2, 3, etc., until computational limits or statistical noise become prohibitive (keeping lx = ly, because your variables are of the same kind). Then compare the results across these parameter settings to assess the robustness of your conclusions.

That said, I recognize that the MD simulations were primarily performed for the contact frequency analysis and that the core findings of the paper are not contingent on the outcome of the transfer entropy analysis. For this reason, I do not consider this issue critical enough to delay publication. If the additional analysis supports your current conclusions, a brief note to that effect would suffice. If, however, the results turn out to be sensitive to parameter choice, the authors should clarify that the transfer entropy results 'suggest' a causal relationship in allosteric modulation, but that further evidence, such as longer trajectories, would be needed to fully rule out memory-dependent effects in MOR dynamics.

We thank the reviewer for this thoughtful and insightful comment and for the constructive suggestions regarding the transfer entropy (TE) analysis. We agree that the assumptions underlying Markov order and delay time merit explicit discussion, and we appreciate the opportunity to clarify our modeling choices and to further assess the robustness of the inferred allosteric communication.

As correctly inferred by the reviewer, our TE calculations employed a first-order Markov process, consistent with the original TE formulations and implementations cited in the literature (e.g., see https://www.biorxiv.org/content/10.1101/170886v1.full or PMCID: PMC5283753), with the delay parameter (τ) set equal to the trajectory saving interval (1 ns). This choice was intentional. Rather than seeking a lag time at which residue dynamics become fully history-independent, our goal was to capture short-timescale, stepwise propagation of conformational information between spatially and functionally proximal residues within the DAMGO- and PAM-bound metastable ensemble. Increasing τ necessarily integrates over multiple dynamical steps and collective motions, which can obscure local residue-level communication pathways that are most relevant for identifying candidate allosteric couplings.

To address the reviewer's concern that residual memory effects could bias TE estimates, we performed robustness analyses by systematically increasing the Markov embedding order while keeping τ fixed at 1 ns. Specifically, we recomputed TE for the dominant residue pairs using second-order ($l_x = l_y = 2$) and third-order ($l_x = l_y = 3$) models. The second-order results are highly consistent with the first-order analysis (Figure H): effective TE values across residue pairs are strongly correlated (Spearman ρ = 0.92), and the identity of the strongest directed interactions is preserved (Jaccard overlap of the top 10% edges = 0.63). This demonstrates that the relative ranking and directionality of dominant information-transfer pathways are robust to inclusion of short-term memory.

H

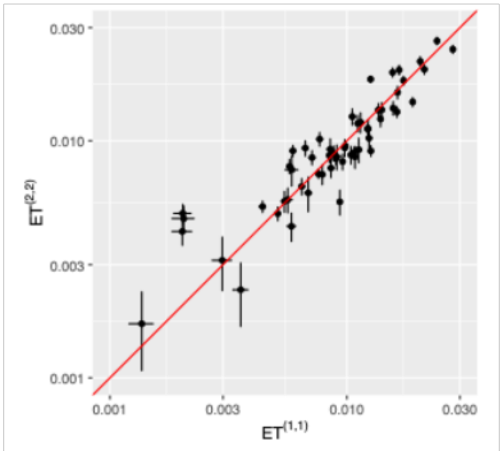

As expected, increasing the embedding order to $l_x = l_y = 3$ substantially amplifies statistical noise due to the exponential growth of the conditional state space and the resulting sparsity of observed histories. Correspondingly, confidence intervals broaden markedly (median SE = 0.0033 for third-order, compared with 0.00028 and 0.00072 for first- and second-order models, respectively), and a larger fraction of edges are not distinguishable from zero. These results indicate that higher-order models do not improve inference at the available sampling depth and instead reduce statistical reliability.

Taken together, these analyses support the use of a first-order Markov approximation at τ = 1 ns as a parsimonious and robust choice for the specific objective of identifying local, directed residue-level communication within the metastable bound state. We have added an explicit description of this robustness analysis to the Methods section (lines 770-776 and 784-801) and note that the dominant TE-derived interactions are insensitive to modest variations in Markov order.

Finally, as the reviewer rightly notes, the central conclusions of the manuscript do not hinge on the TE analysis alone. Rather, TE provides complementary support for the directionality of allosteric communication inferred independently from contact-frequency analysis, mutagenesis, and functional assays.

Author's Response to Reviewers

Reviewer 5 (Remarks to the Author):

In their manuscript entitled "Structural and dynamic studies uncover a distinct allosteric modulatory site at the μ-opioid receptor", Zhang et al. present high resolution structures of the μ-opioid receptor (MOR) bound to the orthosteric agonist DAMGO with and without a cobound positive allosteric modulator (PAM), BMS-986187. Whilst BMS-986187 is a well known and well studied PAM of the opioid receptor family, it's binding site has remained unknown. The structure presented here reveals BMS-986187 binds to a novel allosteric site at the opioid receptor family. Using a combination of mutagenesis, signalling assays combined with molecular dynamics simulations and analysis, the authors provide mechanistic insights into how BMS-986187 allosterically modulates MOR function through this allosteric binding site. The findings are novel to the MOR and wider opioid receptor family and have the potential to aid in the design of novel allosteric modulators for this important therapeutic target.

To improve the manuscript, the following points should be addressed.

Main Points

1. My main concern regarding the Cryo-EM maps is the poor density for the G proteins. One explanation for this is that there is partial occupancy of G protein within the dimer (perhaps due to a clash of the G protein with the extracellular loops of MOR) such that only one of the MOR protomers is bound to G protein, and averaging of this occupancy across the two protomers leads to poor density for the G protein. As the authors state, the anti-parallel dimer species was also observed in previous Opioid Receptor-G protein structures (PMID: 36368306, 36368306). I note that the authors in these manuscripts implemented processing strategies that likely helped in producing high-resolution maps for the entire receptor-G protein complex, strategies which I deduce were not implemented here. For example, PMID: 36368306 performed local refinement with a mask around one G protein, whereas in this manuscript only masks around receptors (monomer vs dimer) were implemented. I recommend trying a similar processing strategies as PMID: 36368306, 36368306 to see if this will improve map quality. Another strategy that may help is to symmetry expand particles using C2 symmetry and then performing 3D classification with the appropriate masks.

Obtaining higher quality density for the G protein will allow the authors to confidently model G protein and allow for expanded discussion on a number of items, including the observed shifts in Gαi1 (line 222) and the PAM induced differences at the MOR-Gαi1 interface (line 244-246).

Without improved density for the G protein, the authors should reconsider depositing models of the entire receptor–G protein complex. Whilst I commend the authors for omitting the AHD domain of the Gα protein due to insufficient density, the density for the RAS domain is also poor and limited. Without improved density, there is currently insufficient data to support modelling of the entire G protein.

We thank the reviewer for the rapid and thorough evaluation of our structural data. We very much agree with the assessment that the cryoEM density for our G proteins is of lower quality, as we had already acknowledged in the results and methods previously: "the corresponding density for the G proteins was of lower resolution" (previous version: Line 119-120), and "we observe considerably lower

map quality for the G proteins compared to the receptor and BMS-986187-binding site" (previous version: Line 619-620).

We appreciate the recommended processing strategies to improve the density, but already attempted improving the density using multiple approaches, including many of those that the reviewer suggested. We have since elaborated on this in the methods: "In contrast to the receptor and ligands, the overall quality of our maps was much lower for the G proteins. We observe good density for about 50% of the Gα Ras domain, the entire Gβ subunit, as well as 50% of the Gγ protein. However, there is little to no reliable density for the parts of the Gα Ras domain and Gγ most distal to the receptor, as well as the Gα alpha helical domain. We unsuccessfully attempted to further improve the maps by i) focused refinement on individual receptor-G protein complexes via fulcrum placement, ii) various 3D classification strategies, iii) using masks to isolate individual receptor-G protein complexes, and iv) refinement without symmetry." (Line 630-637).

We further stated that "We suspect that the lower quality for the G protein is due to asymmetry in the way G proteins are bound in the dimer, and/or lower G protein occupancy with some dimers containing only one G protein." (Line 637-639). This is in response to the author's likely correct hypothesis that the absence of complete G protein density is due to asymmetry or lower G protein occupancy in our structure. In fact, we previously reported structures of the 5-HT1A receptor bound to different G proteins, and observed that only the 5-HT1A-Gz complex formed antiparallel dimers with considerably reduced G protein density (PMID: 40749070). However, in the case of the 5-HT1A-Gz complex, we were able to improve the density by omitting one of the two G proteins in the dimer, a strategy that did not improve the density in the case of our MOR-Gi1 structures.

That being said, we have excellent density for large parts of the three G protein subunits, and feel that a partially built Gα Ras domain and Gγ subunit would be very confusing to readers. After discussing options also with other cryoEM colleagues not involved in this work (e.g. Oliver Clarke, acknowledged in the manuscript) we decided on 2 changes:

1. We now also deposited a receptor-only model for both the PAM-bound and PAM-free structures
2. We will keep the original full MOR-Gi1 structures (minus the AHD), but have set the occupancy values for residues without sufficient density to zero

We now included a justification for this in the main text: "To provide a complete and biologically meaningful representation of the signaling complexes, we nonetheless modeled the heterotrimers in their entirety rather than omitting select regions, but also generated a receptor-only model (see Methods for details)." (Line 120-123). In the methods we have elaborated on this and now state: "Although we obtained lower quality density for the G proteins, the density was sufficient to place Gβ1, and most of Gγ2 and Gαi1 at lower contour levels, though we did omit the Gαi1 alpha helical domain (AHD) entirely. For residues with no discernable G protein density, we, in addition, elected to set the occupancy to zero. Lastly, we also deposited structures where we removed the G proteins from both the BMS-986187-bound and PAM-free complexes. We feel that our overall approach of depositing structures with and without G protein best represents that we have high confidence in our G protein placement, while acknowledging that some areas have poor support from cryo-EM data." (Line 645-653). Lastly, we continue to point out that we refrained from a detailed dissection of potential PAM-mediated effects on the G protein, due to these limitations: "Despite this high degree of similarity, minor shifts in the positioning of the Gαi1 subunits were observed; however, limited G protein

resolution precluded a detailed analysis of potential BMS-986187-mediated transducer rearrangements using cryo-EM data alone." (Line 223-226).

Overall, we feel like we have taken a fairly conservative approach, including disclosing and acknowledging limitations of the structure in multiple sections, as well as curtailing our discussion of G protein structure to a single brief statement about a well ordered region. Models of the receptor-only versions of our PAM-bound and PAM-free MOR structures have now been deposited under PDB codes 10TL (BMS-986187 bound) and 10TM (PAM-free), using the same maps as reported before. This has now also been updated in the Data Availability section in Line 882-889.

Finally, we would like to point out that any discussion about G protein residues with limited density does not affect the findings of our study in any way. Our work is almost exclusively focused on BMS-986187 and its molecular mechanisms, and we have the highest confidence in the density and placement of the PAM.

2. It appears DAMGO is slightly mismodelled with the "F5" moiety of DAMGO positioned outside of what appears reasonable density for this part of the molecule. This is most apparent in the BMS-986187_DAMGO bound structure with the deepEMhancer map (figure uploaded as part of the review). It is less apparent in the DAMGO only structure which is likely due to poorer density for this part of the molecule in the DAMGO only maps. The authors should carefully consider remodelling DAMGO. If ultimately the pose differs between the BMS-986187 bound and unbound structures, a discussion on whether there is an allosteric influence of BMS-986187 on DAMGO's pose is warranted.

We thank the reviewer for spotting this issue. We suspect that this moiety might be fairly flexible as it does not appear to form strong direct contacts with the receptor, and is in an area of the receptor that is easily accessible to solvent. We have now done our best to remodel the F5 moiety of DAMGO to better fit the density, but even the map of the PAM-bound complex has insufficient density to completely fit this moiety. That being said, we were able to move the nitrogen-adjacent Carbon atom into density. After remodeling we do not observe notable differences in the DAMGO binding poses of the PAM-bound and –unbound states, and have therefore no evidence to provide any insight into potential allosteric influence on this region.

3. Beginning line 331, the manuscript contains a sizeable discussion on the rotameric states of Y1513.34 and W1944.50 during the MD simulations of PAM bound and unbound MOR complexes. Yet the manuscript lacks supporting data to visualise these rotameric transitions. To substantiate the claims made in this section, I recommend including figure(s) that directly illustrate the rotameric states of these residues throughout the simulations.

We thank the reviewer for this constructive suggestion. We agree that direct visualization of the rotameric transitions strengthens the discussion. In response, we have now included a new Extended Data Figure 7 that explicitly illustrates the rotameric dynamics of Y151$^{3.34}$ and W194$^{4.50}$ in both PAM-bound and PAM-free DAMGO/MOR–Gi1 simulations. Specifically, the figure provides:

- Time-resolved rotamer assignments (χ dihedral–based classification) across all trajectories after equilibration, enabling visualization of rotameric transitions.
- Population histograms summarizing the percentage occupancy of each rotamer (t, t80, m, m-85, m95) in both simulation conditions.
- Variability across trajectories (shown as standard deviations from bootstrapped sampling).

These analyses directly support the statements beginning at Line 328, which we have further clarified as follows:

- In the PAM-bound system, Y151$^{3.34}$ is strongly restricted to the t80 rotamer, consistent with stabilization by BMS-986187 and the adjacent cholesterol molecule.
- In the PAM-free simulations, Y151$^{3.34}$ samples both t80 and t rotamers with comparable probability, with only minor (~5%) occupancy of inactive-like m-85 and m states.
- W194$^{4.50}$ remains predominantly in the m95 rotamer in both conditions, with only minimal sampling of the m state, despite the progressive weakening of its interaction with BMS-986187.

We have added explicit references to Extended Data Figure 7 in the revised manuscript (Lines 328–340) to ensure that each claim regarding rotameric behavior is directly supported by visual data. In addition, we further corrected several errors related to rotamer terminology (e.g. t80 was frequently named t-80 in the previous version) throughout this section of the manuscript.

We believe these additions address the reviewer's concern and substantially improve the clarity and transparency of the rotamer analysis.

4. A major finding of this manuscript is the 1) central role of W1944.50 in facilitating allosteric transitions at the MOR and the wider opioid receptor family and it's ability to enhance the allosteric effects of PAM's, and 2) the role of cholesterol and the influence that W1944.50 has on cholesterol and the potential interplay between PAM+cholesterol+W1944.50. There are a few points relating to this that are not adequately covered in the manuscript.

- W4.50 is one of the most highly conserved residues in class A GPCRs and its role in facilitating allosteric modulation has been investigated at other GPCRs including the CB1 (PMID: 31659318, 35637350).
- The cholesterol site discovered for the MOR is a well known and well characterised cholesterol binding site at class A GPCRs (PMID: 18547522, 18547522)

A discussion on how the findings at the MOR compare and contrast to these established findings would improve the manuscript.

These are great literature suggestions, and we have since revised our manuscript to include these studies in our discussion. We now state that "Cholesterol is a well-known modulator of GPCR function, and the herein described cholesterol-binding site is a well-known surface that binds cholesterol in several receptors. For instance, studies of the β2 adrenergic receptor suggested that depletion of cholesterol from this site increases inverse agonist affinity, but this likely appears receptor-dependent as

cholesterol depletion at other receptors appears to increase agonist binding (PMID:18547522).” (Line 423-428). We further stated that “Of note, active-like MOR and KOR structures share a unique rotamer conformation of W4.50, providing further evidence that this residue may function as a structural gate during receptor activation. This hypothesis is also supported by previous structural studies of the CB1 cannabinoid receptor: Specifically, the negative allosteric modulator ORG27569 directly binds W4.50, conceivably blocking important activation-related changes in CB1 (PMID 31659318). On the other hand, the PAM ZCZ011 binds in a pocket similar to that of BMS-986187 at MOR, likely allowing for conformational changes in W4.50 (PMID 35637350). That being said, we note that outside of the highly conserved residue W4.50, the surface surrounding this residue is not well conserved between MOR and CB1.” Line (462-471)

5. In relation the the above point, another focus of this manuscript is the differences between BMS-986187 across the MOR, DOR and KOR, and the potential differences in the role for cholesterol in mediating this effect (section beginning line 435). The current discussion of cholesterol's role in this context remains limited and would benefit substantially from newly available structural data. Notably, density for cholesterol molecules has now been observed in the same site at the DOR bound to BMS-986187 and derivatives (recent preprint https://doi.org/10.1101/2025.10.16.682975), contradicting the statement at line 444 that available DOR structures lack a cholesterol molecule at the corresponding site. With these structures now available in the PDB and EMDB, a comprehensive comparative analysis of cholesterol positioning and occupancy with the DOR is now feasible. Such an analysis would substantially strengthen the current discussion—which tentatively suggests that "BMS-986187 may exert subtly distinct modulatory effects at DOR, a possibility that warrants further investigation" (line 441)—by providing direct structural evidence to either support or refine conclusions regarding PAM selectivity and mechanism.

The referenced preprint was published after our manuscript was sent for initial review (notice on 9/1/2025). We usually do not include discussions of work that was published after initial submission of our work, as it invites perpetual cycles of revisions due to requests to discuss data published during revisions. That being said, the referenced preprint is very relevant to our work, and we would be remiss not to at least include a reference to this work in the discussion of our findings. However, there are two closely related issues:

1. It is highly unusual that the DOR structures have been released, even though the manuscript has not been published in a peer-reviewed journal yet. Accordingly, there is no evidence that the released structure has been peer-reviewed, and despite release, the structures may have to be modified prior to publication.
2. The released coordinate files do not contain any cholesterol. In fact, the preprint defines the density in Figure 6g as “potential lipid/cholesterol binding sites”. Assuming these structures could still be under review, what happens if reviewers of the DOR manuscript ask that the DOR cryoEM maps be reprocessed to clarify the role of cholesterol prior to publication in a peer reviewed journal?
   We are therefore concerned that any current analysis requested by the reviewer becomes invalid in the event that the released DOR structures need revision before final publication.

In light of these unusual circumstance we will refrain from analysis of the available DOR cholesterol density, but we have added the following statement in our manuscript: “Of note, a preprint (as of

writing this) reported cholesterol density at DOR nearby the same allosteric site bound by PAMs derived from BMS-986187 (PMID: 41280124). Such work will thus enable a much more comprehensive examination of the differences between cholesterol's role in modulating allosteric activity at MOR and DOR in future work." Line 456-459

Minor Points

1. There is an inconsistency in the reported concentration of BMS-986187 during the purification process. The results section (line 107) states that 100 µM was used, whilst the methods section (line 540) specifies 30 µM. Please clarify/rectify.

We thank the reviewer for catching errors in this section. For the PAM-bound sample, we purified the complex in the presence of 3 µM BMS-986187 as already stated in the methods (Line 545-546), but we did add 100 µM BMS-986187 after purification using the Superdex 200 Increase size exclusion column and before final concentration and grid setup. This has now been clarified in Line 581.

2. Line 297-298. "Unexpectedly, a W1944.50A mutation significantly enhanced the allosteric effects of BMS-986187, both by increasing the PAM's intrinsic efficacy (Fig 4D) and by lowering the concentration required to achieve this effect (Fig. 4A)."

This is two sides of the same coin. If the intrinsic efficacy of BMS-986187 is enhanced at this mutant, then it is expected a reduced concentration of BMS-986187 leads to efficacy.

We agree with the reviewer and have now revised our interpretation to state "Unexpectedly, a W1944.50A mutation significantly enhanced the allosteric effects of BMS-986187 by increasing the PAM's intrinsic efficacy (Fig. 4D)." (Line 297-298)

3. Line 647 states a concentration of 1 nM was used to stimulate cAMP production. This concentation seems rather low. MedChemExpress (the stated supplier) lists an EC50 value of 500 nM of Foskolin for Adenylyl Cyclase. A 1 nM concentration would fall well below the threshold required to produce the suitable increase in cAMP required for observing inhibition via Gi1 activation. Concentrations of 10-100 µM forskolin are routinely used in these assays.

We apologize for this error and thank the reviewer for catching this. We used 1 µM Forskolin, not 1 nM as previously stated. We have now corrected this in the revised manuscript in Line 678.

4. In the figure legends for Figure 1A and Figure 2A, please mention the ligands are shown as sticks and transparent spheres to avoid confusing the transparent spheres for ligand density.

That is an excellent point. So as not to confuse the reader, we have now state that "Ligands are shown as stick models with transparent spheres." in both figure legends.

5. For extended figure 2D, can the authors include which map was used to represent the density?

We have now revised the figure legend of extended data figure 2 to better clarify which map was used for each mesh shown.
